# Abundance and size of hyaluronan in naked mole-rat tissues and plasma

**DOI:** 10.1038/s41598-021-86967-9

**Published:** 2021-04-12

**Authors:** Delphine del Marmol, Susanne Holtze, Nadia Kichler, Arne Sahm, Benoit Bihin, Virginie Bourguignon, Sophie Dogné, Karol Szafranski, Thomas Bernd Hildebrandt, Bruno Flamion

**Affiliations:** 1 Molecular Physiology Research Unit (URPhyM), NARILIS, University of Namur, Namur, Belgium; 2 Department of Reproduction Management, Leibniz Institute for Zoo and Wildlife Research (IZW), Berlin, Germany; 3 Computational Biology Group, Leibniz Institute on Aging–Fritz Lipmann Institute, Jena, Germany; 4 Unit of Methodology and Didactic in Biology (UMDB), NARILIS, University of Namur, Namur, Belgium; 5 Core Facility Life Science Computing, Leibniz Institute on Aging–Fritz Lipmann Institute, Jena, Germany

**Keywords:** Molecular biology, Physiology

## Abstract

Large amounts of ultra-high molecular weight hyaluronan (HA) have been described as the main cause of cancer resistance in naked mole-rats (*Heterocephalus glaber*, NMR). Our work examined HA metabolism in these rodents more closely. HA was localized and quantified using HA binding proteins. Its molecular weight was determined using size exclusion chromatography and gel electrophoresis, HA family gene expression using RNAseq analysis, and hyaluronidase activity using zymography. Guinea pigs (*Cavia porcellus*) and mice (*Mus musculus*) were used as controls for some experiments. We found that HA localization was similar in NMR, guinea pig, and mouse tissues but NMR had larger amounts and higher molecular weight (maximum, around 2.5 MDa) of HA in serum and almost all tissues tested. We could not find ultra-high molecular weight HA (≥ 4 MDa) in NMR samples, in contrast to previous descriptions. Hyaluronidase-1 had lower expression and activity in NMR than mouse lymph nodes. RNAseq results showed that, among HA family genes, *Tnfaip6* and hyaluronidase-3 (*Hyal3*) were systematically overexpressed in NMR tissues. In conclusion, NMR samples, contrary to expectations, do not harbor ultra-high molecular weight HA, although its amount and average molecular weight are higher in NMR than in guinea pig tissues and serum. Although hyaluronidase expression and activity are lower in NMR than mouse lymph nodes, this not sufficient to explain the presence of high molecular weight HA. A different activity of the NMR HA synthases remains possible. These characteristics, together with extremely high *Hyal3* and *Tnfaip6* expression, may provide the NMR with a bespoke, and perhaps protective, HA metabolism.

## Introduction

Hyaluronan (HA) is a glycosaminoglycan (GAG), a polymer of the extracellular matrix made of repeated disaccharide (glucuronic acid/N-acetylglucosamine) units. HA generates different physiological and pathological responses depending on its molecular weight (MW), which varies across different tissues^1,2^. High molecular weight (HMW) HA (> 1 MDa), at least when added exogenously, shows immunosuppressive, anti-inflammatory, and anti-angiogenic effects whereas exogenous low MW (LMW) HA is immunostimulatory, pro-inflammatory, and angiogenic^1,3–5^. The difference may be due to distinct activation of HA receptors or different HA-proteoglycan complexes^6^. LMW HA fragments are produced during matrix remodeling, notably through reactive oxygen species; these HA fragments can amplify immune reactions^6^ or disturb extracellular matrix homeostasis^7^. Inflammation can also induce the production of tumor necrosis factor (TNF)-stimulated gene 6 (TSG6, also known as TNF-α-induced protein 6 or TNFAIP6) and thereby covalent transfer of the heavy chain domains of inter-trypsin α inhibitor (IαI) to HA; this process may alleviate inflammation^8^.

HA undergoes fast turnover. Three HA synthase isoforms (HAS1, HAS2, and HAS3), present in all mammals, synthesize HA at different rates, directly from the inner aspect of the plasma membrane into the extracellular matrix, and with different average sizes. For instance, the medium of mouse *Has1* and *Has3* transfectants contains HA with broad MW ranging from 0.2 to 2.0 MDa, while *Has2* transfectants secrete very large HA with average MW > 2 MDa, possibly around 4 MDa^9^. HAS2 and HAS1 are inducible by inflammatory cues; HAS2 is mostly expressed during development; and HAS3, the most active synthase, is ubiquitously expressed postnatally^5,10^. HA is also constantly degraded (depolymerized) and removed from the tissues. In situ HA degradation accounts for ca 30% while the remaining 70% are drained by the lymphatic vessels to the lymph nodes, where 90% will be degraded and the last 10%, with a reduced average MW, will spill over into the blood and will be taken up mostly by the liver^11,12^.

The principal somatic HA-depolymerizing enzymes, i.e. hyaluronidases, are HYAL1 and HYAL2. HA is degraded at the cell surface by the glycosylphosphatidylinositol (GPI)-linked HYAL2 into intermediate-size fragments. These fragments are internalized and, once in the lysosome, cleaved by HYAL1 to smaller fragments targeted by exoglycosidases^4,5,13,14^. Another hyaluronidase, HYAL3, does not seem to play a direct role in HA catabolism but could increase HYAL1 activity^15^. Furthermore, in mice, *Hyal3* expression increases the expression of *Hyal1*^5,15,16^. In the skin and the osteoarthritic knee^17^, HYBID, also known as KIAA1199 or CEMIP (Cell Migration Inducing Hyaluronidase), has been shown to possess HA-depolymerizing activity. CEMIP belongs to the large family of HA binding proteins (HABPs), which includes CD44, LYVE1 (Lymphatic Vessel Endothelial Hyaluronan Receptor 1), and RHAMM (Receptor for Hyaluronan-Mediated Motility), also known as HMMR (Hyaluronan-Mediated Motility Receptor). An additional transmembrane protein with a sequence related to CEMIP, called CEMIP2 or TMEM2, also controls HA turnover in the matrix^18^ and, when expressed in *Caenorhabiditis* *elegans*, protects this animal from endoplasmic reticulum stress and increases both its longevity and pathogen resistance^19^.

The naked mole-rat (*Heterocephalus glaber*, NMR) is a eusocial^20^, long-lived^21^, tumor-resistant^22,23^ subterranean rodent. In 2013, the NMR was described by Tian et al.^24^ as harboring very high levels of ultra-HMW HA (6–12 MDa) in comparison to laboratory mouse (*Mus musculus*) and guinea pig (*Cavia porcellus*, GP) tissues. These results were obtained using a non-specific Alcian blue staining to assess global HA amount and pulsed-field electrophoresis to assess HA MW. The same study reported that NMR skin fibroblasts in culture secreted 6–12 MDa HA and had lower hyaluronidase activity than human, mouse, or GP fibroblasts. Lower hyaluronidase activity was also observed in NMR tissues. Tian et *al.* claimed the NMR ultra-HMW was the key factor endowing this rodent with its remarkable resistance to tumorigenesis^24^. This claim was based on the observation that the larger MW HA produced by NMR fibroblasts was required to produce enhanced early contact inhibition (ECI), a specific NMR feature leading cells from this organism to stop growing in vitro when they come into contact with each other or with the extracellular matrix. This ECI was inhibited by adding hyaluronidase to the culture medium^24^. Later on, it was shown that the ultra-HMW HA produced by NMR fibroblasts, in contrast to mouse fibroblast HA, exhibited special cytoprotective properties against various stresses^25^. Finally, the HAS2 protein, in NMR showed a unique sequence including two amino-acid changes (2 asparagines replaced by 2 serines) at highly conserved sites responsible for ultra-HMW HA production^24^. *Has2* gene was shown to be overexpressed in NMR skin fibroblasts whereas *Has1* and *Has3* genes did not show any peculiarity.

Surprisingly, the observations on NMR tissue and serum HA, or on ECI, have not been reproduced so far. Our goal was to confirm or amend them, this time using specific and state-of-the-art methods such as histochemistry with a HABP, quantification using an enzyme-linked immunosorbent-like assay, and determination of HA molecular size using size exclusion chromatography (SEC). As a negative control, we performed the same measures in the GP, which is a close relative of the NMR that does not share its tumor resistance and late senescence. In addition, to search for signals of distinct HA metabolism in NMR, we also measured the expression of genes coding for proteins belonging to the broad "HA family" such as HA synthases, hyaluronidases, HABPs, and HA receptors.

## Results

### Alcian blue mainly stains tissue components other than HA

**Figure 1:**
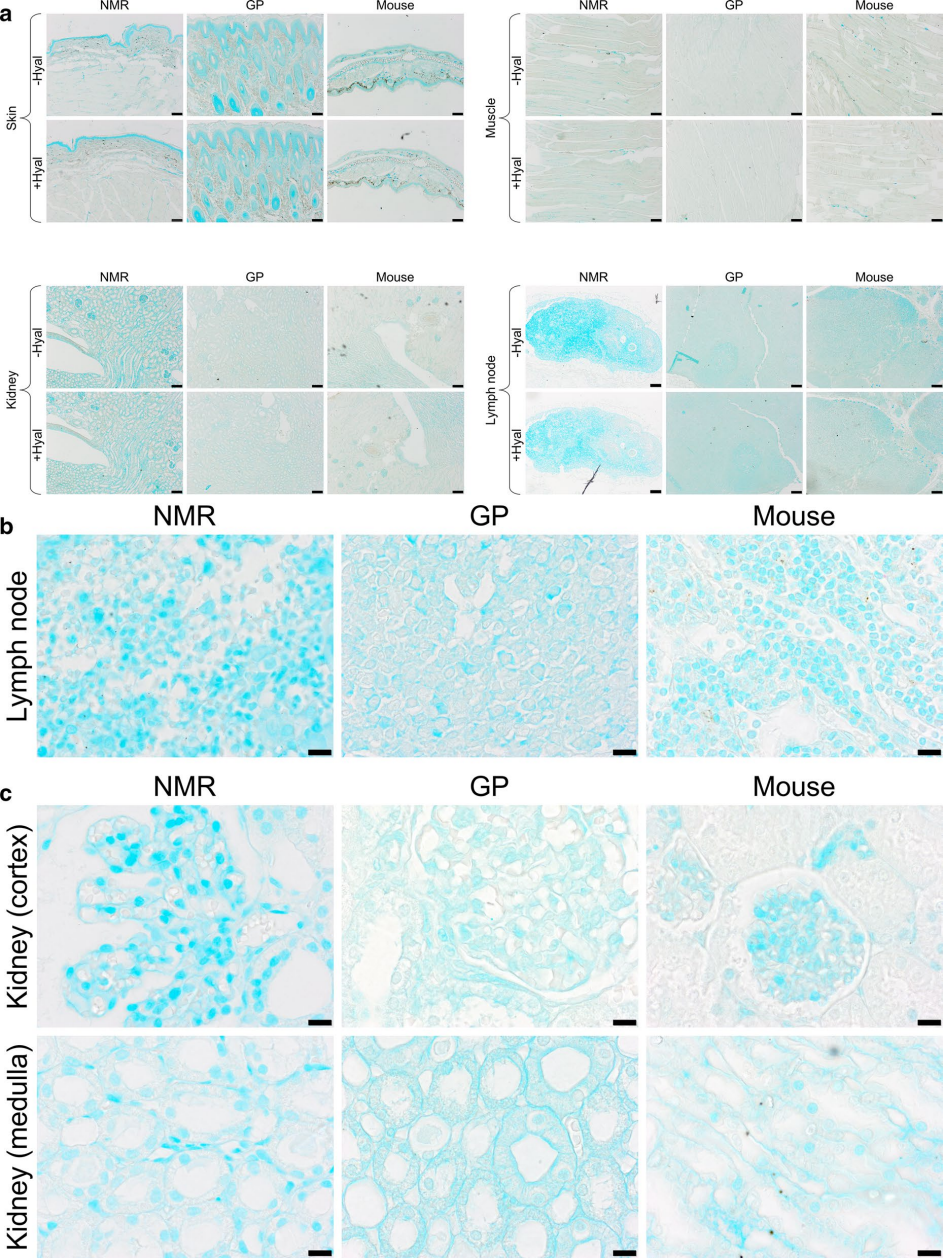
Alcian blue staining of NMR, GP, and mouse tissues. (**a**) NMR, GP, and mouse tissues (skin, muscle, kidney, lymph node) stained with Alcian Blue, with (+) or without (−) 3-h pre-incubation with 100 U/ml hyaluronidase from *Streptomyces hyalurolyticus*. Scale bars, 100 µm. (**b**) NMR, GP and mouse lymph node and (**c**) NMR, GP, and mouse kidney (cortex and medulla) at higher magnification. Scale bars, 10 µm. NMR, naked mole-rat; GP, guinea pig.

**Figure 2:**
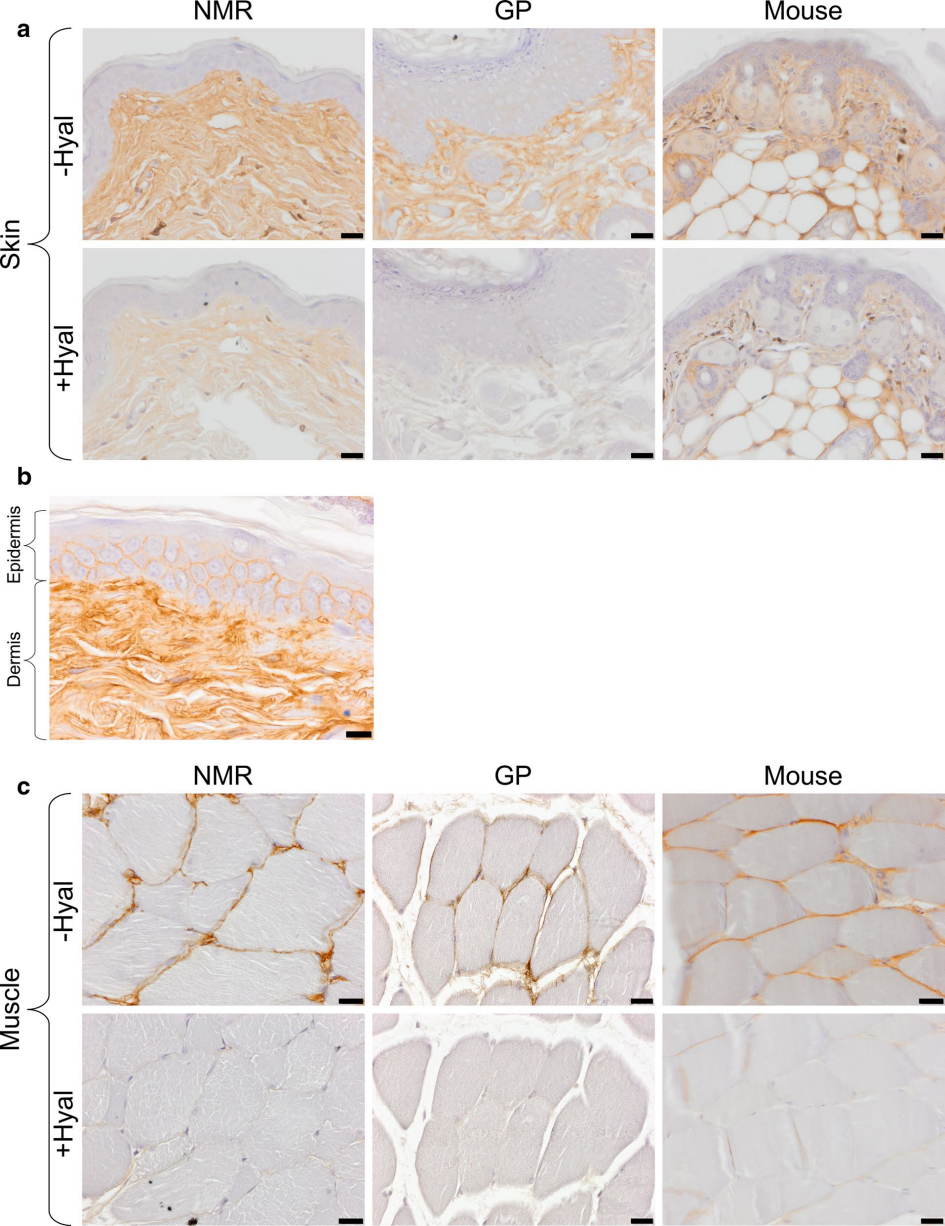

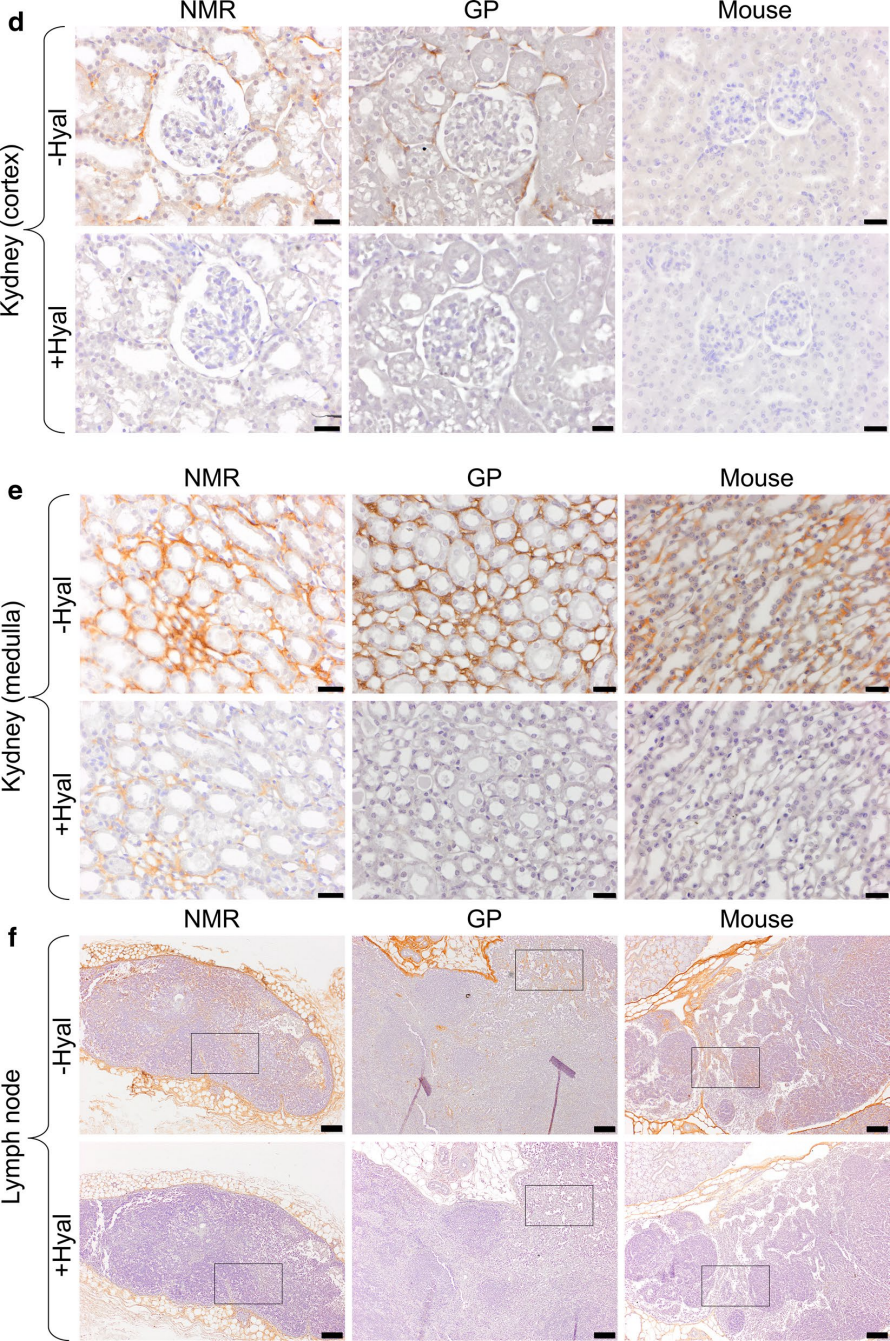

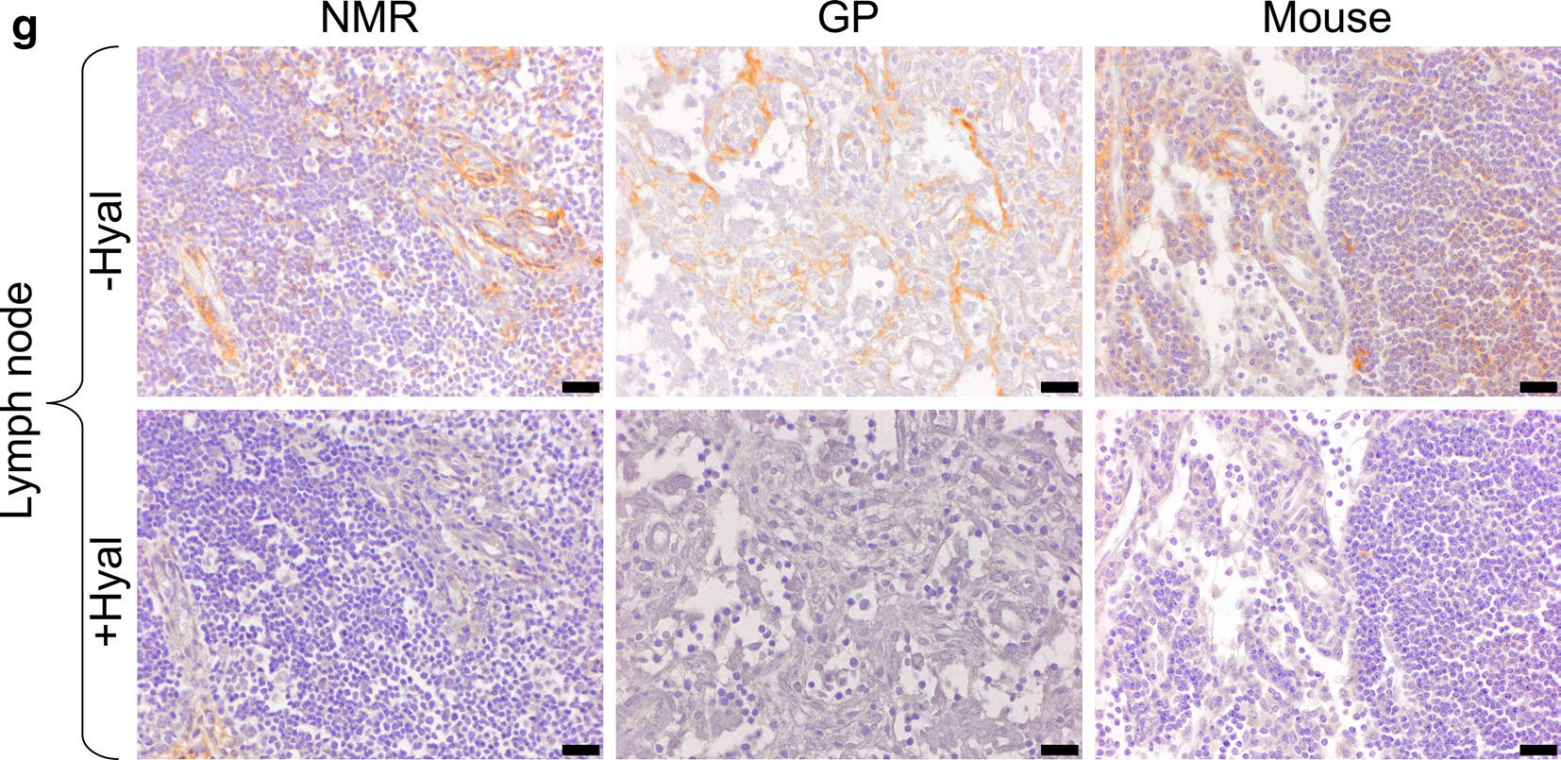
HA staining of NMR, GP, and mouse tissues. (**a**,**b**) skin; (**c**) muscle; (**d**) kidney cortex; (**e**) kidney medulla; (**f**,**g**) lymph node. All tissues were stained using a HA binding protein followed by peroxidase detection, with (+) or without (−) 3-h incubation with 100 U/ml hyaluronidase from *Streptomyces hyalurolyticus*. Positive signals are revealed in brown. Scale bars, 20 µm (**a**,**c**,**d**,**f**), 10 µm (**b**) and 100 µm (**f**). *NMR* naked mole-rat, *GP* guinea pig.

NMR, GP, and mouse tissues were stained with Alcian blue. The results (Fig. 1a–c) show that the staining intensity varies markedly between tissues. It also varies from one experiment to another (data not shown). In most cases, the staining is quite faint and found mostly in connective tissues (e.g., in dermis and muscles); the strongest signals are found in NMR and GP epidermis, NMR renal glomeruli, and throughout NMR lymph nodes. In the latter, at higher magnification, the Alcian blue signal visibly matches nuclei (see Fig. 1b,c). However, in no case does the staining change after HA-selective hyaluronidase pretreatment, the efficiency of which is demonstrated on the same tissues using HABP to specifically label HA (see Fig. 2). That means that, whatever the animal species, Alcian blue mainly stains tissue components other than HA.

### HABP-detected HA localization is similar among the three species

In parallel with these Alcian blue experiments, the same tissues were stained using a HA-specific detection technique, i.e. a HABP and peroxidase revelation method, with (+) or without (−) hyaluronidase predigestion. The results (Fig. 2) show that (a) HA (hyaluronidase-sensitive brown staining) is readily detected in many but not all tissues at varying levels, (b) almost all brown staining disappears during hyaluronidase incubation, and (c) there is no obvious difference in staining in any tissue between the three species, except perhaps, in a minor respect, in the kidney medulla.

As regards HA localization in the skin, the majority of HA is found in the extracellular matrix through the entire thickness of the dermis and sometimes between the keratinocytes in the epidermis (see Fig. 2b). In the muscles, HA is found in connective tissues around the myocytes (basal lamina and endomysium) and around the muscles bundles (perimysium). In the kidney cortex, a light HA marking is present along the epithelial cells (basal lamina) of Bowman’s capsule and in the basal lamina of the tubules around the renal corpuscle except for the mouse, where almost no signal is visible in the cortex. In the kidney medulla, HA is found mainly around the vasa recta, though we cannot exclude that HA also surrounds Henle’s loop. The kidney medulla seems to show a stronger HA staining in GP and NMR vs mouse. In the lymph nodes, HA is found mostly in the connective tissue of the capsule and the trabecula. HA is also present in the connective tissue supporting the trabecular and medullary sinuses. In all tissues, HA is found around the vessels, in the tunica adventitia for arteries and veins, and next to the endothelial cells for capillaries. The HA localization is similar in the three species except in the renal cortex where almost no HA is found in mouse. There are some faint, random differences regarding hyaluronidase sensitivity of the staining among tissues and species.

### Alcian blue staining does not match the HABP-based staining pattern

**Figure 3:**
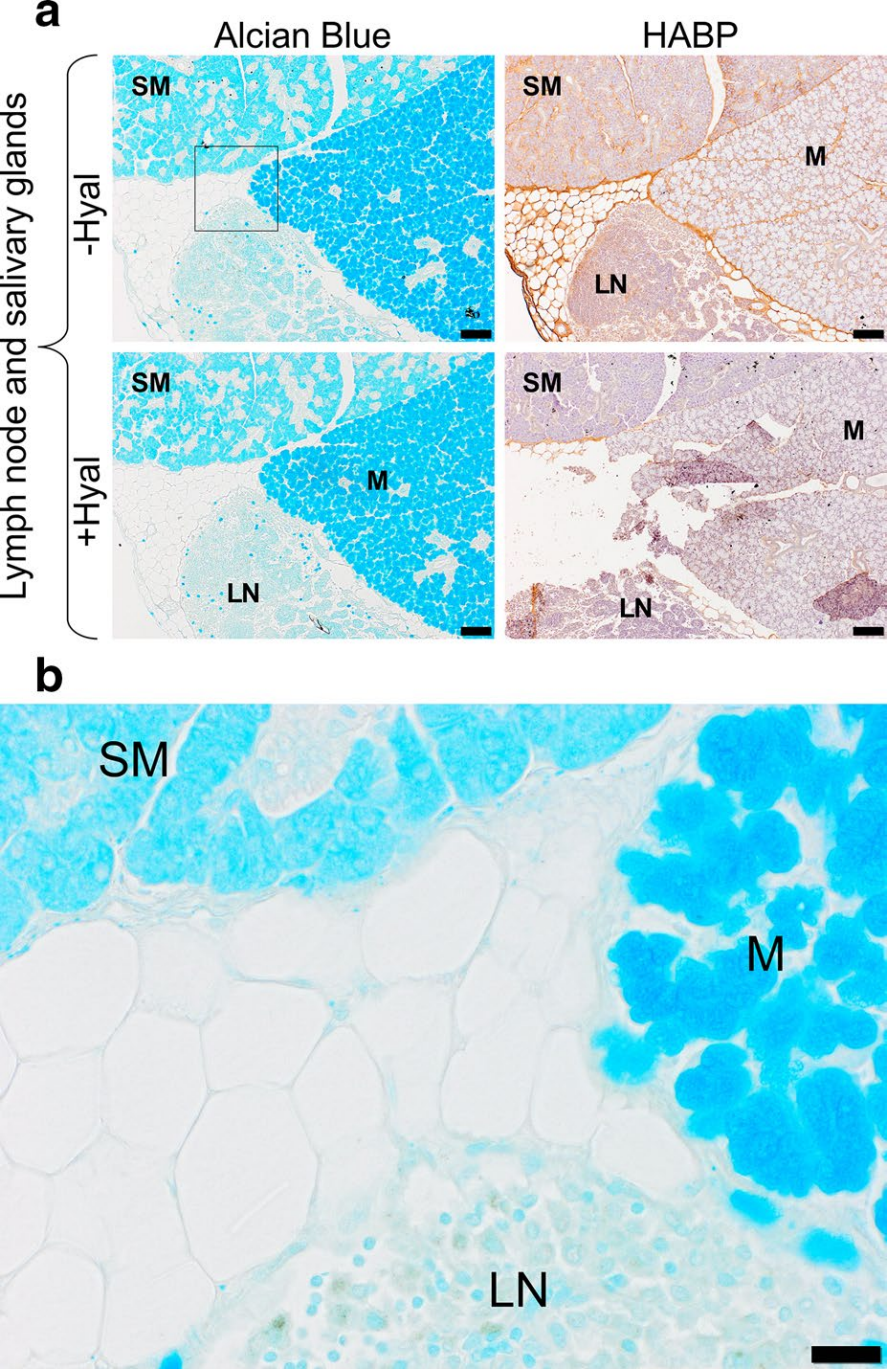
Alcian blue and HA stainings of mouse lymph node and salivary glands. (**a**) Mouse lymph node (LN), and sero-mucosal (SM) and mucosal (M) salivary glands stained with either Alcian Blue or a HA binding protein followed by peroxidase detection, with ( +) or without (−) 3-h incubation with 100 U/ml hyaluronidase from *Streptomyces hyalurolyticus*. Scale bars, 100 µm. (**b**) Alcian blue staining in mouse LN, and SM and M salivary glands at higher magnification. Scale bars, 20 µm.

The HABP-based staining pattern clearly differs from the Alcian blue staining. Because the latter was sometimes faint, we used positive controls (mucosal and seromucosal salivary glands from mouse tissues) to further compare Alcian blue and HABP staining (Fig. 3). The results show that in the salivary glands, Alcian blue intensity is strongest in mucosal glands and stains mucinogen granules inside the cells, whereas HABP-detected HA is found in the connective tissues around the cells and the glands. The blue color intensity ranges from background staining (the lightest blue) in the lymph nodes to more intense staining (the darkest blue) in the sero-mucosal and mucosal salivary glands.

### HA level is higher in most NMR tissues compared to GP tissues

**Figure 4:**
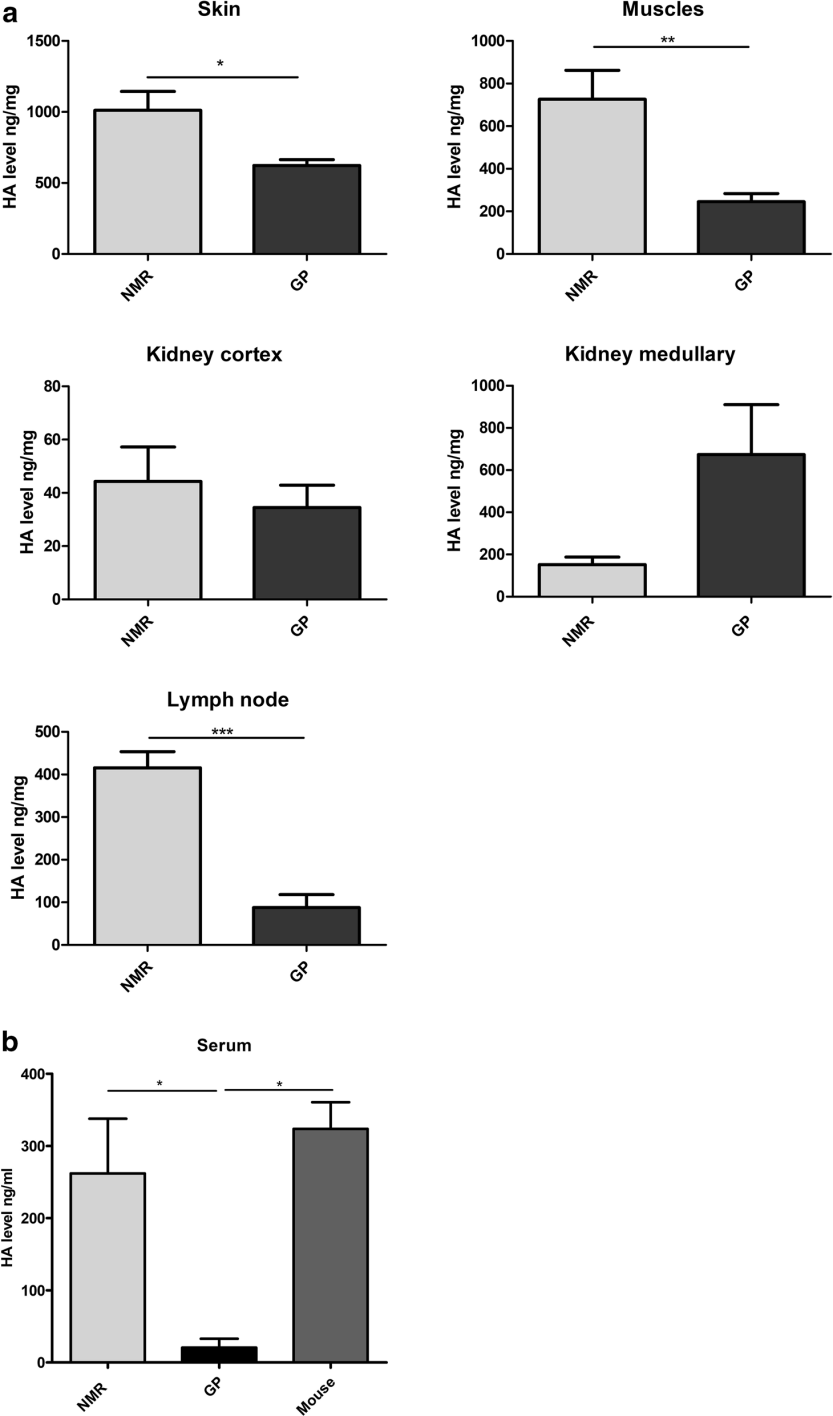
HA level in NMR and GP tissues and in NMR, GP, and mouse serum. (**a**) HA level (mean ± SEM) in NMR (n = 5) vs GP (n = 4) skin, NMR (n = 4) vs GP (n = 5) muscle, NMR (n = 4) vs GP (n = 4) kidney cortex and medulla, and NMR (n = 5) vs GP (n = 4) lymph node. (**b**) HA level in NMR (n = 8), GP (n = 4), and mouse serum (n = 4). Differences between NMR and GP are significant (***, P < 0.001; **, P < 0.01; *, P < 0.05) in skin, muscle, and lymph node (Student's t test), and in serum (ANOVA followed by Holm-Sidak test; the difference between mouse and GP is also significant using that test).

In the next step, HA was extracted and quantified using an ELISA-like assay in the same 4 tissues of NMR and GP (skin, kidney, muscle and lymph nodes), and in the serum of NMR, GP, and mouse. The results (Fig. 4) show that the HA level is systematically higher in NMR organs compared to GP organs (up to 4.8-fold higher in the lymph node), except in the kidney medulla, where the HA level appears higher in GP compared to NMR. In the serum, HA level is > tenfold higher in NMR or mouse than in GP. The NMR and mouse sera appear to contain similar amounts of HA.

### The distribution and peak of HA molecular sizes are shifted toward HMW HA in NMR compared to GP

The next step was to determine the distribution of sizes of HA molecules using SEC in the same tissues (NMR vs GP) and in the serum (NMR, GP, and mouse).

**Figure 5:**
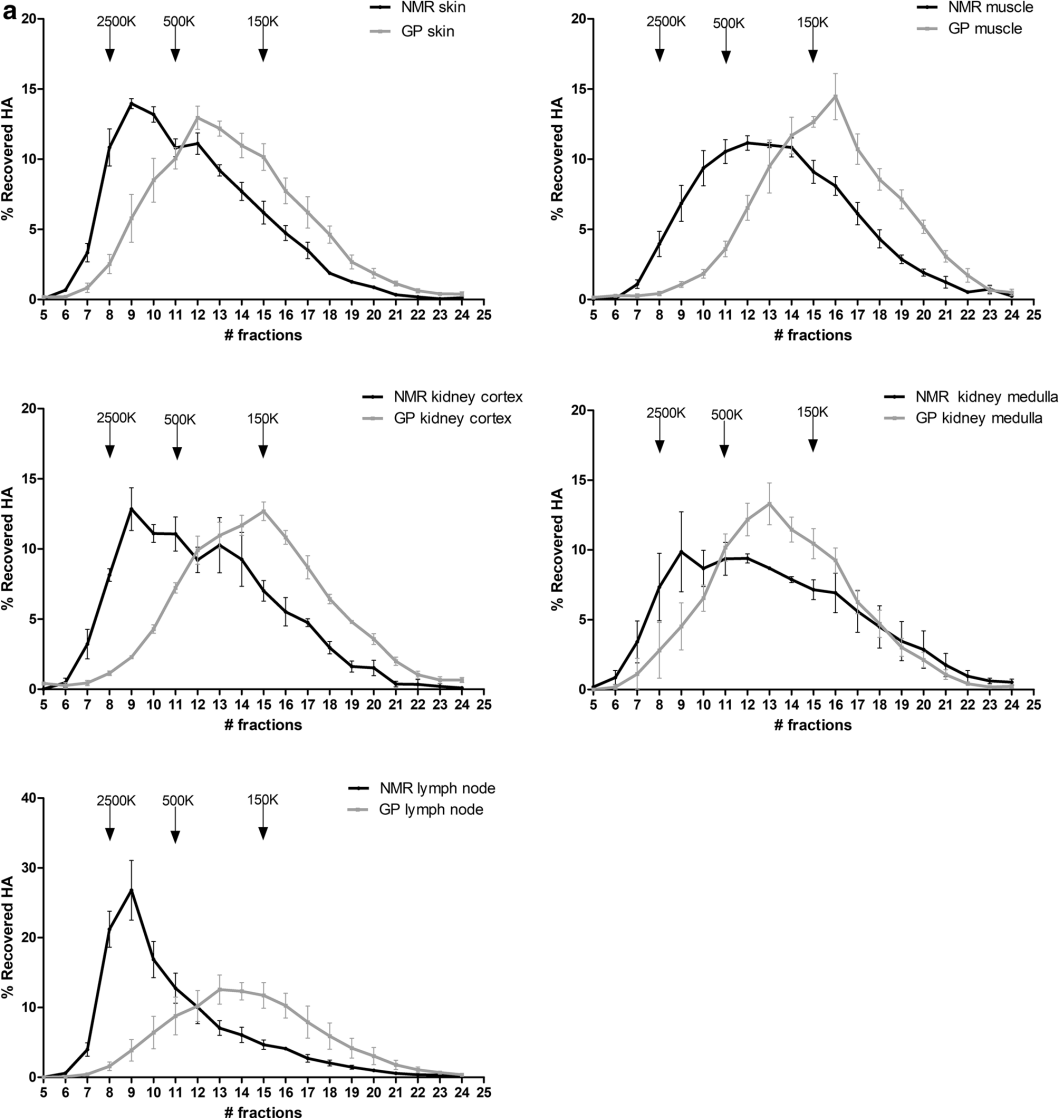

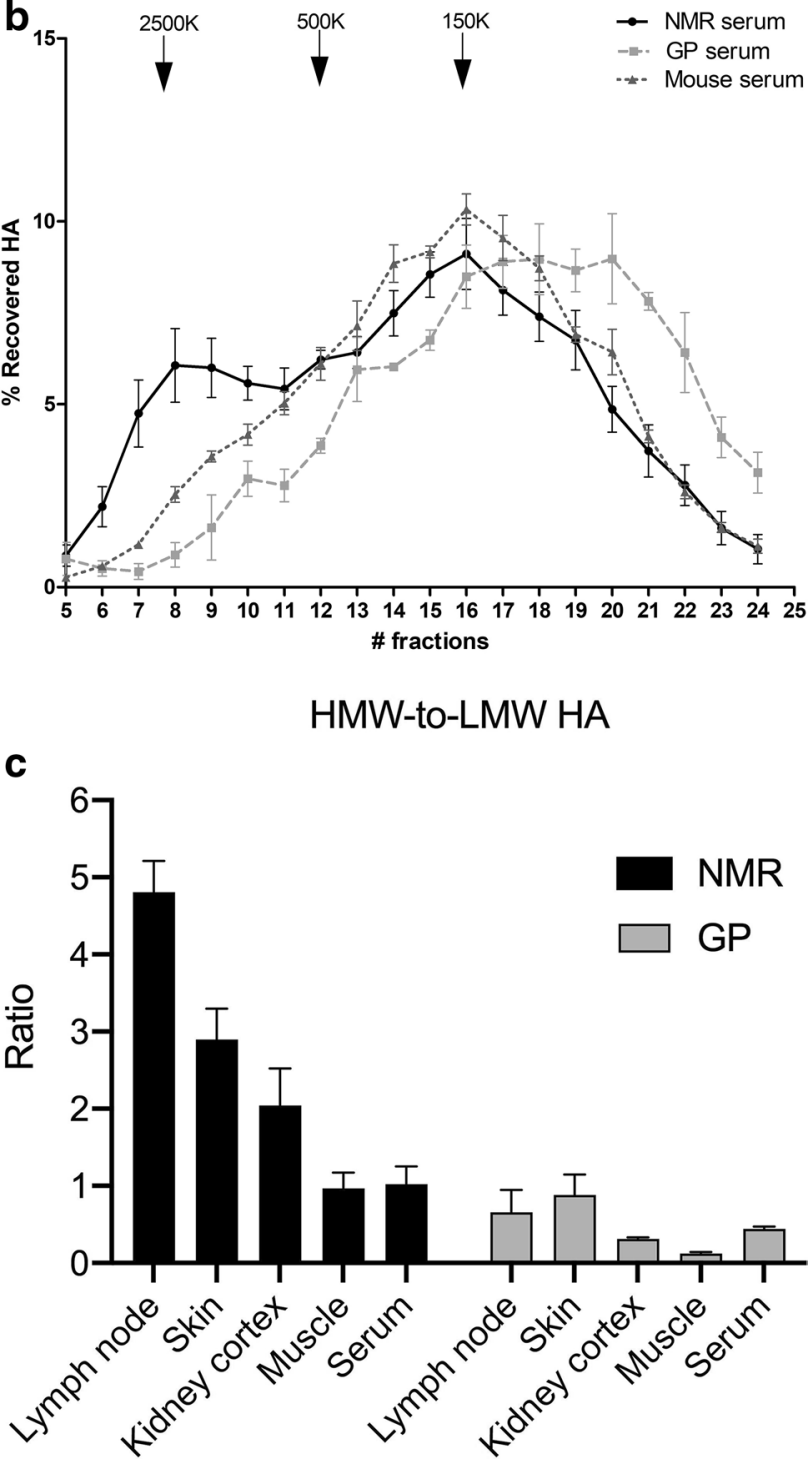
HA molecular weight distribution in NMR and GP tissues, and in NMR, GP, and mouse serum. HA molecular weight in (**a**) NMR (n = 4) vs GP (n = 4) skin, NMR (n = 4) vs GP (n = 4) muscles, NMR (n = 3) vs GP (n = 4) lymph nodes, and NMR (n = 3) vs GP (n = 3) kidney cortex, and NMR (n = 3) vs GP (n = 4) kidney medulla, and (**b**) NMR (n = 11), GP (n = 3) and mouse (n = 4) serum. The elution peaks of three HA standards (2500, 500, and 150 kDa, respectively) are shown by arrows on each graph. (**c**) Ratios of high- (> 500 kDa; HMW) to low- (< 150 kDa; HMW) molecular weight HA in different tissues and the serum of NMR vs GP. Means and SEM are shown.

The results (Fig. 5a) show that the distribution and peak of HA molecular sizes are systematically shifted to the left (higher MW) in NMR vs GP tissues, and, in the serum, for NMR vs GP vs mouse. In the skin, lymph nodes, and kidney, the NMR peak HA MW is somewhat below the standard of 2.5 MDa but clearly above 500 kDa. However, the 2.5-MDa peak makes up most of HA sizes in the lymph nodes, whereas a larger amount of 0.5 to 2.5 MDa molecules are present in the peripheral tissues, skin, and kidney. In muscles, there is no HMW peak; the size distribution is centered around 500 kDa with a symmetrical spread. The GP tissues display a clearly different pattern: there is no HMW peak and the distribution is centered somewhere between 500 kDa (skin) and below 150 kDa (muscles). The difference between NMR and GP is particularly striking in lymph nodes, where the dominant population of HA molecules is of HMW (close to 2.5 MDa) in the NMR and of medium MW (100 to 250 kDa) in the GP.

The serum of the three species (Fig. 5b) contains a majority of medium MW (centered around 150 kDa, in NMR and mouse) or even LMW (clearly < 150 kDa, in GP) HA molecules. The HMW population is still uniquely present in the NMR serum although its proportion is decreased compared to that of the lymph nodes.

These comparisons are summarized in Fig. 5c, in terms of ratios of HMW (> 500 kDa) to LMW (< 150 kDa) HA amounts in different tissues and the serum of NMR vs GP. According to a two-way ANOVA analysis both, the species (NMR vs GP) and the type of tissue are significant factors impacting HMW-to-LMW ratios (p < 0.0001).

### Agarose gel electrophoresis confirms the absence of very HMW HA (≥ 3 MDa) in NMR skin

**Figure 6:**
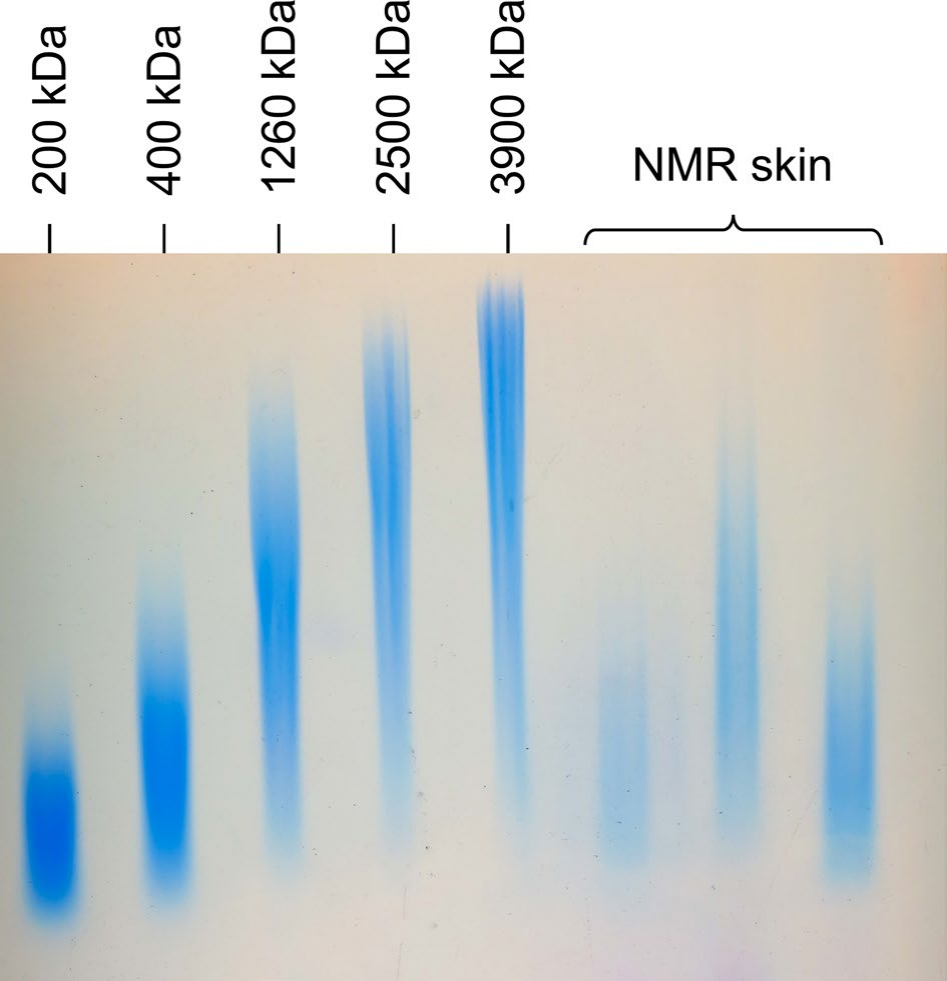
Agarose gel electrophoresis of HA in NMR skin samples. NMR skin samples (n = 3) and HA standards of 200 kDa, 400 kDa, 1260 kDa, 2500 kDa, and 3900 kDa, analyzed using agarose gel electrophoresis and Stains all detection (cropped gel). Full-length blots/gels are presented in Supplementary Fig. 1.

Since SEC did not indicate the presence of significant amounts of ultra-HMW HA, that is, with a MW > 3 MDa, we used another technique, gel electrophoresis, which is not dependent on HA binding proteins, in additional experiments. NMR skin was chosen as a source of HA because of its high amount of HA measured in this study and the fact that the skin was previously described as the tissue with the largest size HA in the NMR (6–12 MDa)^24^. In the current study, agarose gel electrophoresis (Fig. 6) reveals a spread of the MW of HA molecules in NMR skin samples, as compared to standards, between 400 kDa and 1.3 MDa. This result matches the chromatography results. No ultra-HMW HA was detected.

### NMR fibroblasts do not secrete ultra-HMW HA and do not display ECI

**Figure 7:**
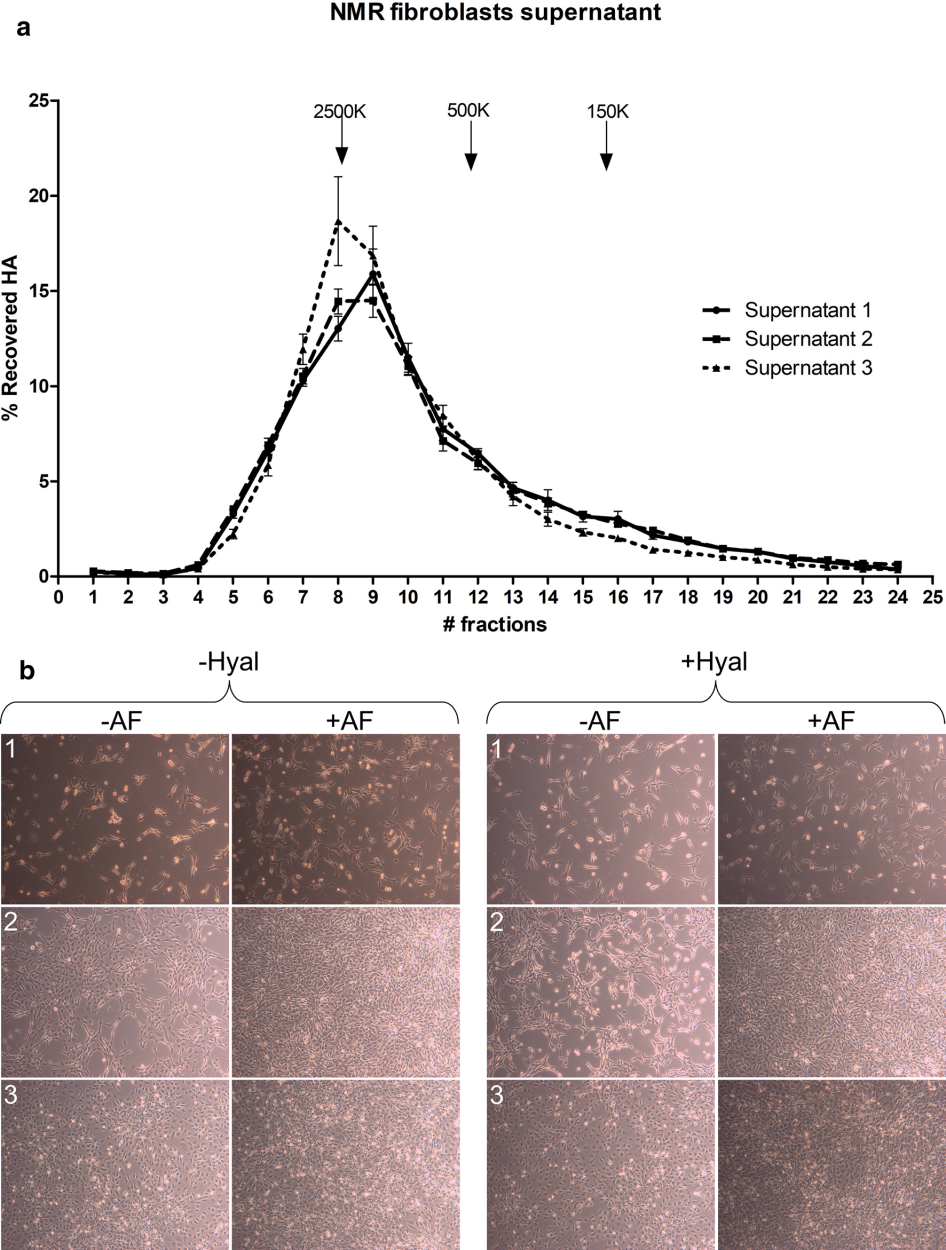
NMR fibroblast culture. (**a**) HA size distribution in NMR fibroblasts supernatant at 3 different times: days 3, 6, and 9, represented by supernatants 1, 2, and 3, respectively (n = 4 cultures for each condition). (**b**) NMR fibroblast culture treated with (+) and without (−) 3 U/ml hyaluronidase from *Streptomyces hyalurolyticus* in the culture media, and with (+) and without (−) attachment factor (AF).

HA size distribution was measured in the supernatant of NMR fibroblasts cultured under natural conditions of low temperature (32.5 °C) and low oxygen content (5%). The results (Fig. 7a) show a size distribution that is similar to that measured in lymph nodes, with a relatively narrow peak centered around 2.0—2.5 MDa. No significant presence of ≥ 3 MDa HA is detected.

In addition, these experiments show there is no clear difference regarding cell confluence with or without hyaluronidase incubation (Fig. 7b). Cells do not show ECI and come to confluence within 9 days; full confluence is reached faster with the use of attachment factor.

### Three HA-related genes (*Hyal3*, *Has1*, and *Tnfaip6*) are highly overexpressed in NMR vs mouse lymph nodes

**Figure 8:**
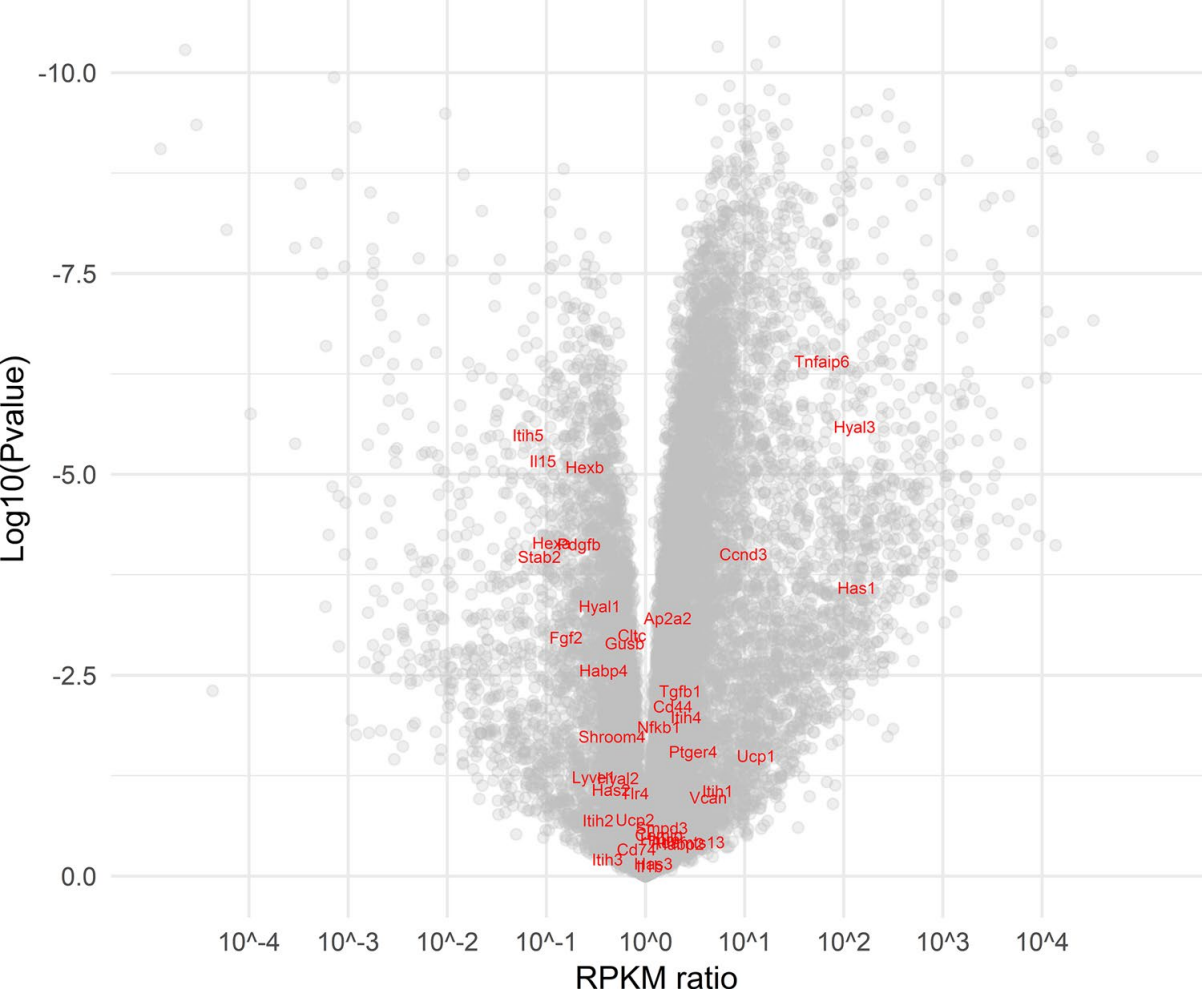
HA-family gene expression in NMR vs mouse lymph nodes. Volcano plot of HA-family genes, spotted in red on grey background of all other genes. The p-value for differential gene expression between the two species is plotted vs gene expression fold-change.

Considering the role of lymph nodes in HMW HA degradation and the marked differences in HA amount and size between NMR and GP lymph nodes (see Figs. 4a and 5a), we decided to analyze the expression of 38 HA-related genes, i.e., those genes coding for proteins with HA-synthase, hyaluronidase, or HA-binding properties, using RNA sequencing of NMR vs mouse lymph nodes (GP lymph nodes were not available). We visualized the results with a volcano plot approach. A volcano plot is useful to detect the most highly upregulated (towards the right hand side of the plot) or downregulated (towards the left hand side of the plot) genes with the highest statistical significance (towards the top of the plot). As shown in Fig. 8, none of the HA-family genes is found on the top of the plot (left- or right-hand sides) which means that they are not among genes in lymph nodes that show significant inter-species differences.

If we look more precisely at HAS and hyaluronidase genes (Table 1), we discover that *Has2, Hyal1,* and *Hyal2* expressions are 2- to 3-fold higher in mouse than in NMR, whereas *Hyal3* is highly overexpressed (129-fold) in NMR vs mouse (Fig. 8 and Table 1). *Has1* and *Tnfaip6* (the latter codes for a modifier of HA interactions in the extracellular matrix) are also found to be highly overexpressed (100-fold and 58-fold, respectively) in NMR vs mouse lymph nodes (Fig. 8 and Table 1).

**Table 1:** HA-family gene expression in NMR vs mouse lymph nodes.

	NMR	Mouse
*Has1*	2.09 ± 0.64	0.022 ± 0.01
*Has2*	0.76 ± 0.28	1.58 ± 0.32
*Has3*	1.06 ± 1.21	0.61 ± 0.10
*Hyal1*	0.64 ± 0.20	1.86 ± 0.49
*Hyal2*	7.49 ± 2.94	13.41 ± 3.18
*Hyal3*	24.96 ± 15.93	0.19 ± 0.02
*Tmem2*	14.254 ± 4.43	11.510 ± 3.28
*Tnfaip6*	36.86 ± 6.97	0.64 ± 0.10

Expression in RPKM (mean ± SD of 7 selected HA-family genes (*Has1, Has2, Has3, Hyal1, Hyal2, Hyal3* and *Tnfaip6*) in NMR (n = 4) and mouse (n = 3) lymph nodes.

### *Hyal3* and *Tnfaip6* genes are overexpressed in NMR tissues compared to GP tissues

**Figure 9:**
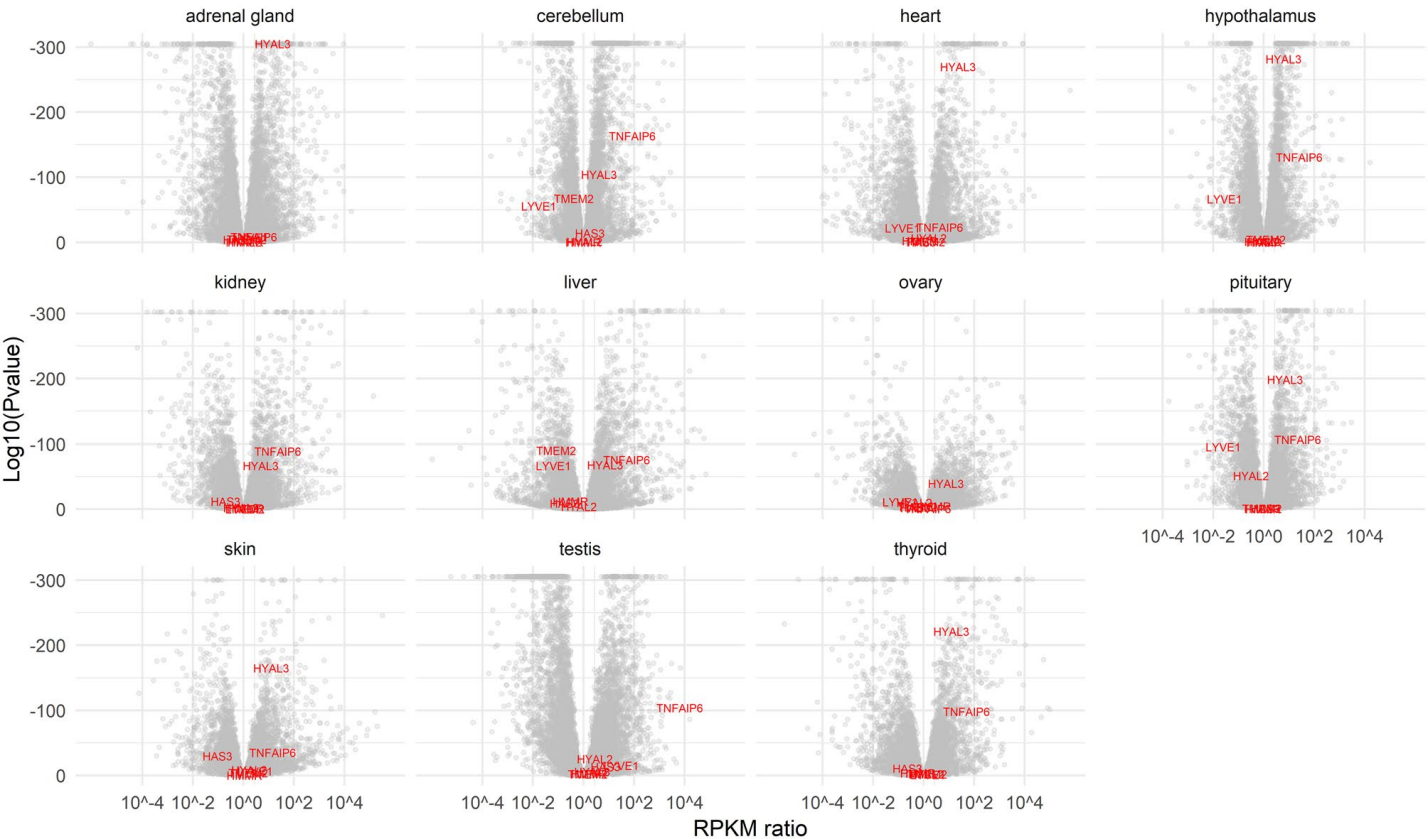
HA-family gene expression in NMR vs GP. Volcano plot of 6 HA-family genes (*Has3, Hmmr, Hyal2, Hyal3, Lyve1, Tmem2* and *Tnfaip6*), spotted in red on background of all other genes in grey, representing on y-axis, the statistical significance (log(P value)) vs, on x-axis, the magnitude of change (RPKM ratio) between NMR vs GP gene expression in 11 tissues (skin, heart, hypothalamus, testis, ovary, kidney, liver, thyroid, pituitary gland, cerebellum, adrenal gland).

To determine whether specific HA-family genes are differentially expressed across tissues in the species comparison NMR vs GP, a published RNAseq data set^26^ was used, but only 7 HA-family genes could be further analyzed. These 7 genes did not comprise, for instance, *Has1* or *Hyal1*, due to significant sequence differences in the assembled NMR vs GP transcripts. A cross-tissue p-value was calculated for each of the 7 genes: Only 2 of them, *Hyal3* and *Tnfaip6*, are significantly different between the two species at cross-tissue level (p-value < 0.01). Both have a much higher expression in NMR compared to GP (Fig. 9).

### HYAL1 activity shows a unique pattern in NMR lymph nodes and serum compared to mouse and GP

**Figure 10:**
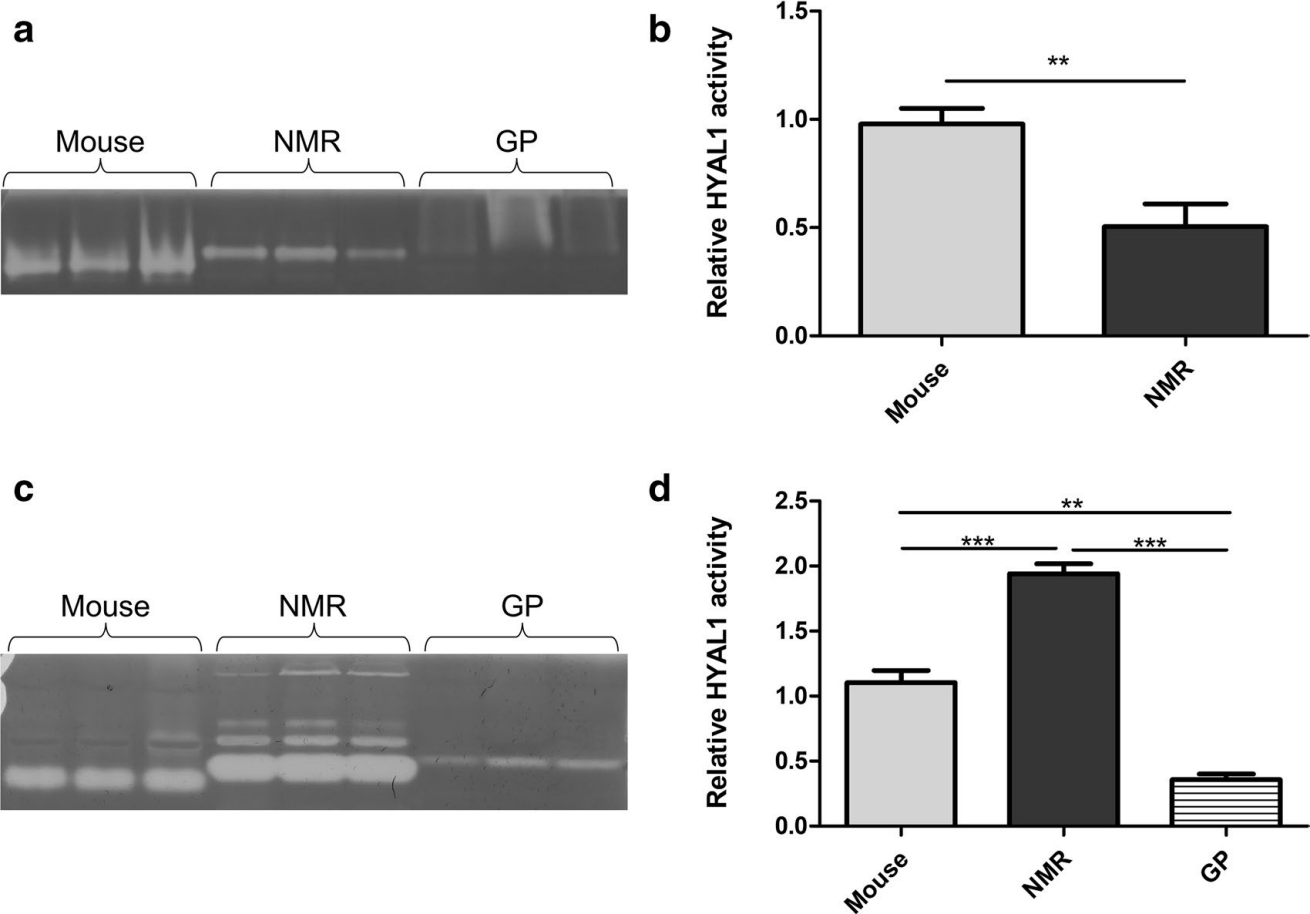
HYAL1 activity in NMR, GP, and mouse lymph nodes (**a**,**b**) and serum (**c**,**d**). HYAL1 activity was measured using native zymography in mouse, NMR, and GP (**a**) lymph nodes, and (**c**) serum (cropped gels). Relative HYAL1 activity was assessed in (**b**) lymph nodes (n = 3), and (**d**) serum (n = 3). Means ± SEM are shown. Differences are significant (***P < 0.001; **P < 0.01; *P < 0.05) in mouse vs NMR lymph nodes (Student’s t-test; no detectable activity was found in GP lymph nodes), and between the three species in serum (ANOVA and Bonferroni test). Full-length blots/gels are presented in Supplementary Fig. 2A,B.

HYAL1 activity was explored in NMR, GP, and mouse lymph nodes and serum using native protein zymography. The results (Fig. 10a,b) are consistent with the RNAseq data and show that the relative activity of HYAL1 is significantly higher in mouse than in NMR lymph nodes (and possibly in other tissues). In the serum (Fig. 10c,d), a completely different pattern emerges, in that HYAL1 activity is much higher in the NMR serum compared to the mouse and GP. However, due to the neutral pH, HYAL1 cannot be active in the serum itself.

## Discussion

Our study was designed to evaluate HA localization, amount, and MW in NMR tissues and serum, since the species has been described as harboring ultra-HMW HA^24^, with sizes (6–12 MDa) that are at the highest level ever described in any animal or human tissue to date. That ultra-HMW HA has been proposed as an endogenous anti-aging and anti-cancer agent^24,25,27^. However, so far the localization and amount of HA in NMR tissues have been determined only by using a non-specific dye, Alcian Blue, so that it became crucial to confirm these initial observations using more selective techniques of detecting and measuring HA. The current study has used state-of-the-art techniques to this aim and has compared the NMR to the phylogenetically related, the GP. Some data were also obtained from C57BL/6 mouse.

Our histochemical results, illustrated in Fig. 1, show that the intensity of Alcian blue staining in both NMR and GP varies between tissues, is generally faint, and does not disappear with HA-specific hyaluronidase incubation. Our results show that Alcian Blue mainly stains tissue components other than HA, including nuclei. These results clearly differ from those of Tian et al.^24^, most likely because Alcian blue is not HA-specific. By targeting acidic groups^28–30^, Alcian blue is known to stain all GAGs, i.e. heparan sulfate, chondroitin sulfate, dermatan sulfate, keratan sulfate, and HA, as well as the large family of mucins found, for instance, protection and lubrication of the salivary glands^31–33^ (Fig. 3). It has also been shown that Alcian blue can stain cell nuclei with variable specificity^29,34,35^. There is no way to make Alcian blue staining a specific detection method for HA^32,36–40^. In the current study, we have followed a protocol very similar to that described in Tian et al.’s study for Alcian blue staining^24^. Possible minor differences are formalin concentration (4% in our study vs 10% in Tian et al.'s study), time spent in fixative solution, potential change in HA structure during tissue manipulation (although unlikely), and hyaluronidase concentration and duration of incubation. It is unclear if these differences could explain the large discrepancy in Alcian blue staining between the studies.

Whatever the cause of the discrepancy between our Alcian blue results and those of Tian et al.^24^, we have not been able to detect a much stronger HA staining in NMR vs GP using the recognized HA specific marker, HABP (See Fig. 2a–g). The utilization of HABP is now widely accepted as the only specific method to localize HA in tissues^41^. Our results in mouse tissues show a very similar HA pattern (mainly in connective tissues) compared to the pattern described previously in that species. Briefly, HA is mostly localized around the sinuses of the lymph nodes, in the interstitial space of muscle cells, and in the connective tissue of the skin^13,42^. We found a similar distribution in the NMR. Our results in NMR and mouse skin are also similar to those previously described in human^43^ and mouse dermis^13^ and epidermis^44–46^. In NMR skin, Kulaberoglu et al.^47^ have recently found larger HA immunofluorescence signal detection in NMR vs mouse epidermis (around the keratinocytes). However, the global HA skin staining appear similar in both species (their Fig. 1a,b). In the kidney, our results show a very similar HA localization between NMR, GP, and mice tissues (except for the mouse kidney cortex where HA is almost not visible) with no clear increase in the staining of NMR tissues. It has been shown that even with a 1.5- to threefold increase of the amount of HA, there is no visible difference in terms of HA staining through HABP in the same tissues^13^.

Regarding the amount of HA in tissues, the general HA content profile appears to be similar between NMR and GP, i.e., HA content of skin > muscles > lymph nodes > renal medulla > renal cortex, and similar to the HA content profile described in mice^13,48^. The absolute amounts of HA in GP and mouse tissues appear similar^13^, although cross-study comparisons of absolute HA values should be treated with caution due to large variations between measurement methods and their local application. However, NMR tissues have clearly higher HA content (except for the renal medulla) than GP skin, muscles, and lymph nodes, confirming previous results^24^ regarding a relative HA enrichment in NMR tissues, even if the measurement techniques differed (See Fig. 4a). The only exception is the renal medulla, in which HA content appeared lower in NMR than the other species. HA content in the mouse and rat renal medulla is known to be much higher than in the cortex and to increase during water loading^49,50^. The role of this interstitial HA is likely to reduce water reabsorption and allow the excretion of diluted urine, as shown by the situation of gerbils (desert rodents), a species with extremely high urinary concentrating ability, that exhibit very low papillary HA content, and even lower content during water loading, to ensure maximal water conservation^51^. The renal medulla average HA content we found in the NMR (150 µg/g dry weight) is closer to that of desert gerbils (150 µg/g dry weight in published data [59]) than to those of GP (670 µg/g dry weight in this study). However, caution should be exercised regarding historical comparisons of HA content, and we do not know how the NMR renal medullary HA content would respond to water loading. The NMR natural environment is in arid and semi-arid zones but with quite stable humidity conditions (close to saturation) leading to a probably low evaporative water loss. These conditions are replicated in captivity. The NMR has no access to free water but shows only a moderate, not very high, kidney concentrating ability^52^. We can surmise that life in high humidity conditions does not put a high stress on the urine concentrating powers of this animal. Further interpretation of its relatively low medullary HA content would require the results of a water loading test.

The NMR serum HA level (260 ng/ml) is close to that of the mouse (See Fig. 4b), as measured in this (~ 320 ng/mL) and previous studies (300 ng/ml^53^ to 600 ng/ml^54^). These values are also in the same range as those measured in other species like dog, pig, goat, sheep, cow, and rat, i.e., between 100 and 700 ng/mL^55^. The levels described in humans are usually lower, i.e., around 40 ng/mL^55,56^ but may reach 200 ng/mL^53^. The most likely explanation for the very low levels found in GP serum in this study (~ 20 ng/mL) is that the ELISA-like assay used produces lower signals for < 20 kDa HA molecules, which are abundant in GP serum^57^. Low values of HA levels have also been reported in rabbits and in some rat studies^55,58^. An additional observation of our study was a high variability in NMR serum HA levels, ranging from 44 to 650 ng/mL in a sample of n = 8 animals. One possible explanation could be variable diurnal lymph flow in the NMR, perhaps linked to bursts of physical activity, as has been described in other species and in humans^59^. It is also known that HA content raises with age, including studies on humans^55^; therefore, it would be interesting to monitor NMR serum HA levels across their lifetime.

One of the main goals of the current study was to determine the distribution and average MW of HA molecules in NMR tissues. For that we turned to FPLC-SEC, a method with high precision and reproducibility^60^. SEC coupled with ELISA-like assay is widely used to determine HA MW because it is both specific and sensitive^1,61^. Agarose gel electrophoresis is another ordinary and robust technique to measure HA size^1^ but it requires HA-rich samples allowing loads of at least 7 µg of HA per well^62^. Electrophoresis can tolerate impurities but those impurities can be stained and can impair the HA size measurement^1^. Therefore, considering the quantity and purity of HA available per tissue sample, we decided to use the FPLC-SEC coupled with ELISA-like assays on all tissues and serum (See Fig. 5a,b), together with agarose electrophoresis (See Fig. 6) only on the tissue richest in HA, the skin.

Our SEC results show, without any doubt, that the size of HA in NMR tissues is systematically higher than in GP tissues. The decreasing order of magnitude of HA sizes runs from skin and lymph node to kidney and muscle; the same ranking was previously observed in mouse tissues^13^. The highest NMR HA size is close to, but not higher than, 2.5 MDa in skin and lymph nodes, as confirmed by agarose electrophoresis on skin samples. The size range of NMR HA molecules in situ is similar to that of the mouse, except for lymph nodes, which contain a sharp peak centered around 2.5 MDa in NMR as opposed to a smooth and flat peak between 500 kDa and 2.5 MDa in the mouse. Because significant amounts of lower MW HA are present in NMR peripheral tissues (skin, kidney, muscles), the lymph node profile suggests most of the removal of lower MW HA is performed in the tissues themselves and does not travel through the lymphatics. Future studies could determine if specific or unusual HA removal systems exist in NMR tissues.

The presence of a large HMW HA peak in lymph nodes, a part of which spills into the serum, is unusual but not unheard of. A similar peak has been observed in transgenic HYAL2 knockout mice, suggesting a unique role of HYAL2 in getting the lymphatic fluid rid of HMW before this fluid reaches the blood^13^. One explanation for the NMR lymph node and serum profiles could thus be a very low activity of HYAL2 in the lymph nodes. However, this activity cannot be measured in situ based on current knowledge and methodology; it can only be assessed in isolated cells (e.g.^63^). The RNAseq data obtained in our study seem to rule out a markedly decreased transcription of the *Hyal2* gene, but we have no information about NMR lymph node HYAL2 activity. Tian et al.^24^ described a generally reduced hyaluronidase activity in the NMR tissues and cultured fibroblasts but the distinction between HYAL1 and HYAL2 was not possible and the lymph nodes were not directly examined.

In summary, our results confirm a higher average HA size in NMR than in GP and mouse peripheral tissues and serum, and constant exposure of NMR tissues and blood to a high proportion of HMW HA compared with LMW HA (See Fig. 5c). Because of the well-known divergent properties of HMW vs LMW HA^1,6^, this difference between NMR and GP may have an enormous impact on phenomena such as inflammation, aging, and possibly oncogenesis.

On the other hand, we were unable to detect any relevant amount of HA molecules larger than 2.5 MDa, even in the supernatant of fibroblast cultures (See Fig. 7), as opposed to what Tian et al. described in 2013^24^ and Takasugi et al. in 2020^25^. We have no direct explanation for the discrepancy, except highlighting the specificity and sensitivity of the methods we used. Tian et al. used pulsed-field electrophoresis, a technique that was developed to separate DNA molecules up to 2000 kb but has not been well characterized in terms of its ability to separate HA molecules without increasing their apparent size. As already discussed above, one remote possibility to explain the discrepancy is based on HA’s ability to change conformations under particular circumstances^64,65^ in association with the specific HA folded structures and assemblies in NMR tissues^47^. We have kept the treatments done to the tissues, cells, and supernatants at the minimum, whereas other authors have resorted to purification procedures that may modify the HA structure^24,25,47^ and induce HA binding to other HA molecules or to other GAGs with an increase in its apparent MW and viscosity. Whether a similar explanation could apply to the observation of ECI in cultured NMR fibroblasts by Tian et al.^24^ but not in our experiments is unknown. However, other authors failed to observe resistance to oncogenic transformation in NMR fibroblasts^66^.

The high amount of 2.5-MDa HA in the NMR lymph node extracts suggests a particular activity of HASs, hyaluronidases, or HABPs in that organ. When measured using RNAseq, the HA-related gene family as a whole was not drastically modified in NMR vs mouse lymph nodes. It is unlikely that the 2- to threefold lower expression of *Hyal1* and *Hyal2* could explain a large increase in HMW HA in the lymph nodes since that anomaly has only been found in a full *Hyal2* knockout mouse, and not even in the Hyal1 knockout mouse^13^. Similarly, the striking overexpression of *Has*1 in NMR lymph nodes is unlikely to explain the difference in HMW HA, since HAS1 is not known to synthesize HA molecules with MW > 2 MDa^9,10^. There is thus no clear explanation for the high amount of 2.5-MDa HA in the NMR lymph node extracts. On the other hand, RNAseq data revealed two other large up-regulation of HA-related genes: *Hyal3* and *Tnfaip6* (coding for TSG6), at 129- and 58-fold vs mice, respectively.

HYAL3 is considered an inactive enzyme of the hyaluronidase family^14–16^. However, in human cells and tissues, an mRNA spliced variant of *HYAL3* can regulate global hyaluronidase activity, mostly determined by HYAL1^67^. It was even suggested that the full-length form of HYAL3 (but not the alternatively spliced forms) may be enzymatically active^67^. Although more recent studies were unable to confirm any direct HA-degrading activity of HYAL3^14,16^, they described a clear link between *HYAL3*/HYAL3 and *HYAL1*/HYAL1. For instance, in vitro overexpression of *HYAL3* increased the levels of *HYAL1* mRNA, HYAL1 protein, and hyaluronidase activity^15^. Conversely, in *Hyal3* null mice, *Hyal*1 expression was decreased by 60% in liver cells^16^, and in parallel, in *Hyal1* null mice, the liver *Hyal3* transcripts were significantly enhanced^68^. Thus, a feedback loop may exist between *HYAL3*/HYAL3 and *HYAL1*/HYAL1. HYAL3 has therefore been proposed as either a partner for stabilizing or folding HYAL1 during its intracellular processing, or an intracellular HABP helping to increase HA uptake and transport to the lysosome^15^.

Based on these elements, we measured HYAL1 activity in NMR vs GP and mouse lymph nodes (See Fig. 10a,b). In line with a double expression of *HYAL1*, we found an almost doubled level of HYAL1 activity in mouse vs NMR lymph nodes. Therefore, the high expression of HYAL3 mRNA in NMR does not lead to a higher activity of HYAL1, at least in the lymph nodes. Based on knowledge obtained in HYAL1 knockout mice, the reduced activity of HYAL1 in NMR tissues is unlikely to explain the higher average MW of HA in the NMR. The zymography performed to measure HYAL1 activity also revealed visible differences in level of migration between the NMR, mouse, and GP proteins. These differences might result from different net charges for these 3 glycoproteins, or from their association in multiprotein complexes of different size and/or charge that would migrate at different speeds in the native gel. Surprisingly, in serum, HYAL1 activity is lower in mouse and GP than in NMR (See Fig. 10c,d). The serum pH is not optimal for HYAL1 activity^69^, so the “native zymography” results (experiments conducted at pH 3.5) do not reflect the fully effective activity of the native precursor conformation, knowing that this precursor is also able to hydrolyze HA^70^. Since HYAL1 is an intracellular enzyme^70^, we could hypothesize that HYAL1 in mouse and GP is mostly trapped inside cells while NMR HYAL1 may display a greater extracellular release. Though not formally tested, the hypothesis of an increased secretion of HYAL1 in the presence of high expression of HYAL3 cannot be excluded, since *Hyal3* knockout mice have much lower expression of HYAL1 in the liver, the main organ releasing HYAL1 into the bloodstream^16^. The results in GP are even more surprising because in all cases, the HYAL1 activity is low. The clearance of HYAL1 by the liver of the various species remains an open question. In a nutshell, the link between *HYAL3*/HYAL3 and *HYAL1*/HYAL1 in NMR does not seem to follow the same “rules” as those previously described in other species, and should thus be explored in more details.

TSG6 is a HA-binding protein with a key role in inflammation as well as in ovulation. TSG6 is induced by progressive inflammation and, conversely, reduces inflammatory cues^8^. It has been demonstrated that TSG6 can interact with HA and deeply change the HA network conformation, leading to the covalent link of HA with the heavy chains of Inter-α-Trypsin Inhibitor involved in tissue protection, HA receptor interactions, and pathological situations^71^. Conversely, HA can induce TSG6 oligomerization^72^.

Knowing that chronic inflammation can act as an aging risk factor and contribute to age-related diseases^73^ and that in contrast, NMR shows a negligible senescence phenomenon^74^, it can be hypothesized that TSG6 is involved in the extended lifespan of NMR by controlling inflammation. An alternative hypothesis is that *Tnfaip6* is constantly induced by repeated inflammatory bouts in the NMR but this seems less likely. TSG6 can also influence the bind between HA and CD44^75,76^. The TSG6•HA complex can switch CD44 into an active state and enhance or inhibit HA-CD44 binding. When TSG6 is present in excess, which might be the case in NMR, the HA-CD44 link is inhibited because TSG6 has more affinity for HA than CD44. When the TSG6/HA ratio is reversed, the binding of CD44 to TSG6•HA is augmented^75^. We can even go one step further and suggest that the high TSG6 levels of NMR reduce cellular stress transduced by CD44 and p53 and protect all NMR tissues against aging. In fact, it was recently shown that very-HMW HA molecules (> 6 MDa) bind CD44 and thus reduce the binding of CD44 with other proteins, leading to a partial attenuation of p53 and its target genes, whereas "simple" HMW HA (~ 2.5 MDa) has the opposite effect and facilitates the damaging p53 downstream effects^25^. In that study, the CD44-p53 axis was involved in major types of cellular stress, such as oxidative stress. The link between HA, CD44, and TSG6 could be the basis of a dynamic and unique mechanism in NMR cells and tissues. This hypothesis remains to be tested.

## Materials and methods

### Tissue and serum sampling

Male and female NMRs between 0.5 and 5 years of age from the Leibniz-Institute for Zoo and Wildlife Research [Leibniz-IZW; Berlin, Germany] colony were used in all experiments. Tissue samples collected from the skin, leg muscles (quadriceps), lymph nodes (from the mediastinal, axillary, and popliteal areas), and kidneys were either rapidly frozen in liquid nitrogen (for HA amount and MW determination) or fixed in 4% formalin (for histology). Freshly collected lymph node samples were dipped into RNA Later (Qiagen) for RNA analyses. Blood was collected, centrifuged, and the serum was rapidly frozen in liquid nitrogen. Male and female GPs of 1 year of age (Dunkin Hartley HsdDhl:DH, Harlan Laboratories, AN Venray, Netherlands) were raised in the Leibniz-IZ. Tissue samples were collected and processed in the same way as for the NMR. Serum from male and female GPs (Dunkin Hartley strain) of 12 to 18 weeks of age was recovered from whole blood samples by KyvoBio SRL (Evere, Belgium). Tissue and serum samples from male and female mice **(**C57BL/6 strain) between 4 and 7 months of age were obtained from the University of Namur colonies and processed as those of NMR and GP.

### Skin fibroblast culture

Cryopreserved skin fibroblasts obtained via skin explant culture from a 3.1-year-old female NMR (5th passage) were thawed and cultured in 35-mm Petri dishes at NMR body temperature (32.5 °C) and low oxygen pressure (5% O_2_, 5% CO_2_) with DMEM yellow (DMEM/F12 + GlutaMAX) and 15% FBS (Sigma-Aldrich, St. Louis, MO, USA), 1% penicillin–streptomycin, and 1% Antibiotic–Antimycotic (Thermo Fisher Scientific, MA, USA). Cells were plated in wells pretreated or not with Attachment Factor Protein (1X) (Thermo Fisher Scientific, MA, USA), and cultured either with or without sterile-filtered 3 U/ml (final concentration) hyaluronidase from *Streptomyces hyalurolyticus* (Sigma-Aldrich, St. Louis, MO, USA). Controls of each culture condition were performed without addition of cells. The medium was changed every 3 days; at the same time, microscopic pictures were taken, and 400 µl of supernatant were collected of each culture condition, immediately frozen, and stored at − 80 °C until further analysis.

### HA quantitative assay (sandwich assay)

Tissues were weighted before being lyophilized. Dry tissue samples were weighted again and digested for 24 h at 55 °C with 3 mg/ml protease (Pronase E, P5147; Sigma-Aldrich, Bornem, Belgium). The samples were diluted in PBS containing 0.05%Tween-20. HA concentration was quantified using Hyaluronan DuoSet kit (R&Dsystems, Abingdon, United Kingdom) according to the manufacturer’s instructions.

### Histological staining

Fixed and paraffin embedded tissues were sliced into 6-µm thick sections, which were deparaffinized in toluene, methanol 100%, and methanol 70%, successively, and rehydrated in tap water. Sections were finally stained, fixed, and photographed with a light microscope (Olympus BX63, Cell Sens software).

#### HA Peroxidase detection

HA was labeled in tissue sections using biotinylated HABP (Calbiochem, San Diego, CA, USA). Briefly, sections were deparaffinized and rehydrated. After incubation with 3% hydrogen peroxide for 5 min at room temperature to block endogenous peroxidase activity, sections were then incubated with 0.2% bovine serum albumin and 0.02% tritonX-100 in PBS for 30 min at room temperature. Before HABP incubation, controls were incubated for 3 h at 37 °C with 100 U/ml hyaluronidase from *Streptomyces hyalurolyticus* (Sigma-Aldrich, St. Louis, MO, USA). All sections were then incubated 2 h with HABP at 1.6/100 dilution with 0.2% bovine serum albumin and 0.02% tritonX-100 in PBS. HABP staining was achieved with a 1/100 strepavidin-HRP (Perkin Elmer, Massachusetts, USA) solution for 30 min at room temperature and 3,3′-diaminobenzidine (Liquid DAB + substrate Chromagen System; Dako, Santa Clara, CA, USA) for color development. Finally, sections were counterstained with hematoxylin (Sigma-Aldrich, St. Louis, MO, USA) and mounted using DPX (HA staining in brown).

#### Alcian blue

Before Alcian blue staining, controls were performed using 3-h incubation at 37 °C with 100 U/ml hyaluronidase from *Streptomyces hyaluronidase*. The slides were then incubated with 1% Alcian blue 8GX (Thermo Fisher Scientific, MA, USA), pH 2.5, microwaved for 5 min at 80 W, followed by an additional 10 min incubation in the warm Alcian blue solution, and then washed three times in distilled H_2_O, dehydrated and mounted with DPX.

### HA size distribution

#### Size exclusion chromatography

Tissue HA concentration was measured following lyophilization, treatment with Pronase to eliminate contaminants, and reconstitution in PBS-Tween as described above. A predetermined amount of tissue extract was analyzed using FPLC (Fast protein liquid chromatography)-SEC (size exclusion chromatography) on a Sephacryl S-1000 (GE Healthcare Europe GmbH, Diegem, Belgium) glass column (length 26 cm, diameter 0.6 cm). Samples were eluted at 70 µl/min with 0.15 M sodium acetate, 0.1% 3-[(3-cholamidopropyl)dimethylammonio]-1-propanesulfonate hydrate (CHAPS; Sigma-Aldrich, St. Louis, MO, USA). Thirty fractions of 200 µl each were collected. Controlled size HA samples of 2500, 500, and 150 kDa (SelectHA; Hyalose LLC, SanDiego,CA, USA) were used as standards. HA concentration was measured in each fraction and expressed as a percentage of the total HA recovered.

#### Agarose gel electrophoresis

Tissue HA concentration was measured as described above. Samples were prepared in the same way as for SEC. A predetermined amount of tissue extract was analyzed using agarose gel electrophoresis. Fourty-four µl of each sample was mixed with 6 µl of 2 M sucrose loading solution. Then samples were separated on 0.8% agarose gel (Promega-Madison, WI, USA) for 11 h at 60 V in TAE buffer and then, stained with 0.005% Stains-all overnight at room temperature. The size of the HA molecules was determined by comparison with HA clinical solutions of 200, 400, 1260, 2500, and 3900 kDa (Pharmacia, Stockholm, Sweden).

### RNA sequencing and differential gene expression analysis

For RNA isolation, tissues were suspended in the homogenization buffer of the RNA extraction protocol and then disrupted using a Tissue Lyser instrument (Qiagen). RNA was isolated using the RNeasy Mini kit (Qiagen). The RNA was treated with DNase I on the affinity column before elution. Library preparation, including poly-A( +) mRNA selection, was done using Illumina's TruSeq RNA Library Prep Kit v2 kit following the manufacturer's description. Quantification and quality check of the libraries was done using Agilent's Bioanalyzer 2100 in combination with a DNA 7500 Kit (both Agilent Technologies). Sequencing was done on a HiSeq 2500 running the machine in 51-cycle, single-end, high-output mode by multiplexing seven samples per lane. Demultiplexing and extraction of read information in FastQ format were done using the bcl2astq tool (v1.8.4; provided by Illumina). RNA-seq reads were mapped with TopHat2 (v2.1.0) and BOWTIE (v1.1.2) using the appropriate Ensembl gene annotation in conservative mode and requiring unique hits for each read. Read counts were derived using feature Counts (v1.5.0) and values normalized to fragments per kb transcript per million fragments (FPKM) as described in previous studies^26,77^.

For differential gene expression analysis of NMR vs mouse tissues, 14,807 genes were analyzed and 38 HA-related (“HA-family”) genes were spotted. These genes were *Adamts13, Ap2a2, Ccnd3, Cd44, Cemip, Cltc, Fgf2, Gusb, Habp2, Habp4, Has1, Has2, Has3, Hexa, Hexb, Hmmr, Hyal1, Hyal2, Hyal3, Il1b, Il15, Itih1, Itih2, Itih3, Itih4, Itih5, Lyve1, Nfkb1, Pdgfb, Ptger4, Ptx3, Shroom4, Stab2, Smpd3, Tgfb1, Tlr4, Tmem2, Tnfaip6/Tsg6,* and *Vcan*. Three selected genes (*Cd74, Ucp1,* and *Ucp2*) were used as tissue markers.

For the NMR vs GP comparison, raw data were obtained from a previous study^26^. The previous study listed fold-differences for about 9600 homologous genes in 11 tissues (skin, heart, hypothalamus, testis, ovary, kidney, liver, thyroid, pituitary gland, cerebellum, adrenal gland). Gene expression was measured only on transcript fragments that were highly similar between NMR and GP, and files were constructed to list fold changes and p-values. Of these 9600 genes, only seven HA-family genes were found at a detectable level and with sufficient sequence similarity: These seven genes were considered for further analysis: *Has3*, *Hmmr*, *Hyal2*, *Hyal3*, *Lyve1, Tmem2,* and *Tnfaip6*. In order to aggregate a cross-tissue p-value *p* from the given data per gene $g$, we used Fisher's method test statistics ${F}^{g}$ modified by weight^78^:

$${F}^{g}=|\sum_{t=1}^{l}({\mathrm{log}}_{e}\left({p}_{g}^{t}\right)*{w}_{g}^{t})|$$

with ${w}_{g}^{t}=sgn({logFC}_{g}^{t})$, where ${p}_{g}^{t}$ and ${logFC}_{g}^{t}$ are the p-value and logarithmized fold-change, respectively, for the gene with index $g$ and tissue with index $t$; $sgn$ is the signum function, and $l$ is the number of examined tissues. We empirically estimated the null distribution of the test statistic by 100,000 resampling rounds using the original 9600 genes. To aggregate a cross-tissue p-value for the differential expression of the total of the 7 HA-relevant genes, an unweighted Fisher's method test statistic $\mathcal{F}$ was used:

$$\mathcal{F}=\sum_{g=1}^{6}{\mathrm{log}}_{e}\left({\rho }^{g}\right)$$

where ${\rho }^{t}$ is the cross-tissue p-value for the gene with index $g$.

### HYAL1 activity assay

The activity of HYAL1 was measured using the technique of ‘native protein zymography’. The lymph nodes and serum samples were first prepared in lysisSDS-free sample buffer (saccharose 0.25 M and triton 1%) and then in SDS‐free sample buffer and resolved in a SDS‐free polyacrylamide gel. Next, the gel was successively incubated in 0.1 M formate buffer, pH 3.7 containing 0.1 M NaCl for 30 min at RT, and then for 20 h at 38.5 °C in fresh formate buffer to allow Hyal1 to degrade the HA included in the gel. After washing with distilled H_2_O, the gel was incubated for 2 h at 38.5 °C in 40 ml Tris/HCl, pH 8.8 containing 0.1 mg/mL of Pronase (Type IX from *Streptomyces griseus*), then washed in distilled H_2_O. Finally, the gel was incubated in 50% formamide for 30 min at RT prior to staining with a 0.015% Stains all (Eastman Kodak Company) 50% formamide solution for at least 1 day in the dark. The gel was discolored in distilled H_2_O and photographed on a transilluminator.

### Ethics committees

NMR and GP: Animal housing and tissue collection at the Leibniz Institute for Zoo and Wildlife Research was compliant with national and state legislation. Breeding allowance (#ZH 156) and ethics approval (G 221/12; T 0073/15) were granted by the local Ethics Committee of the Regional Office for Health and Social Affairs Berlin, Germany (Landesamt für Gesundheit und Soziales, Berlin, Germany).

#### Mice

Animal breeding and experiments were conducted in accordance with the national (Belgium) and local (University of Namur) animal ethics committees.

### Statistical tests

Graphical representations and statistical analyses were realized using GraphPad Prism software (GraphPad Software, La Jolla, CA, USA). Mean values ± SEM were used for data representation. Comparisons between NMR and GP (or mouse) were assessed using Student's t-test (see Figs. 4a and 10b) or a two-way ANOVA for multiple comparisons between tissues; one-way ANOVA followed by either Holm-Sidak test (see Fig. 4b) was used to compare NMR and mouse vs GP assessments or Bonferroni test (see Fig. 10d) was used to compare NMR vs mouse vs GP assessments. Values of P < 0.05 were considered to represent significant differences from the null hypothesis. Graphical representations of volcano plots were realized using R and RStudio.

## Supplementary Information

Supplementary Information
