# Supplementary material for: Abundance and size of hyaluronan in naked mole-rat tissues and plasma
Source: Sci Rep. 2021 Apr 12;11:7951. doi: 10.1038/s41598-021-86967-9 (PMC8041917; doi:10.1038/s41598-021-86967-9)
Supplement: Supplementary file 1 — Supplementary Information [file 41598_2021_86967_MOESM1_ESM.docx]

Abundance and size of hyaluronan in naked mole-rat tissues and plasma

Short Title: Hyaluronan in naked mole-rat

Delphine del Marmol^1*^, Susanne Holtze^2^, Nadia Kichler^2^, Arne Sahm^3,^ Benoit Bihin^4^, Virginie Bourguignon^1^, Sophie Dogné^1^, Karol Szafranski^5^, Thomas Hildebrandt^2^, Bruno Flamion^1^

^1^Molecular Physiology Research Unit (URPhyM), NARILIS, University of Namur, Belgium

^2^Leibniz Institute for Zoo and Wildlife Research (IZW), Department of Reproduction Management, Germany

^3^Leibniz Institute on Aging –Fritz Lipmann Institute, Computational Biology Group, Jena, Germany

^4^Unit of Methodology and Didactic in Biology (UMDB), NARILIS, University of Namur, Belgium

**Supplementary Information**

**Supplementary figure 1**

**
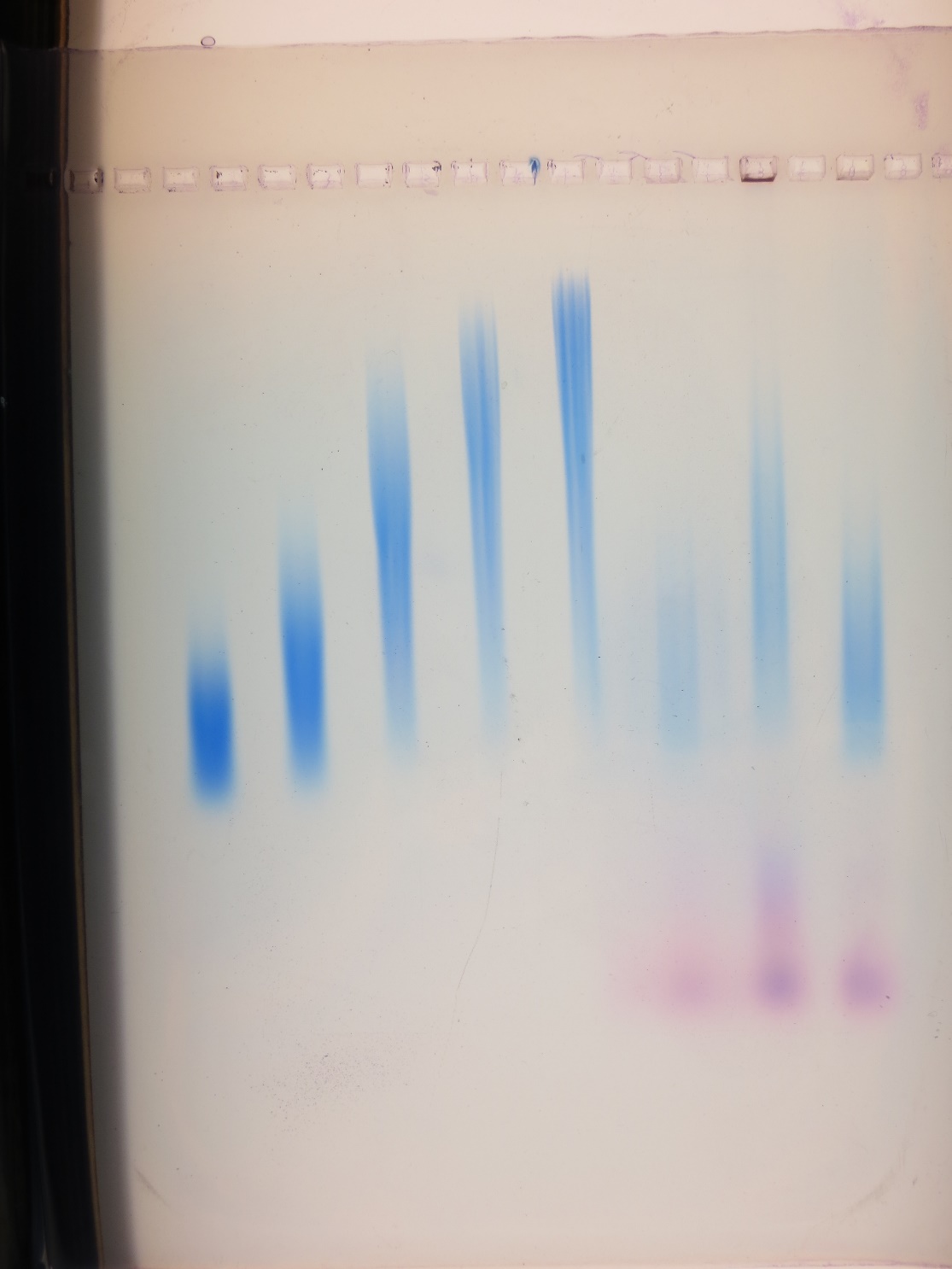
**

**S figure 1: Agarose gel (full-length) electrophoresis of HA in NMR skin samples.**

NMR skin samples (n=3) and HA standards of 200 kDa, 400 kDa, 1260 kDa, 2500 kDa, and 3900 kDa, analyzed using agarose gel electrophoresis and Stains all detection.

A


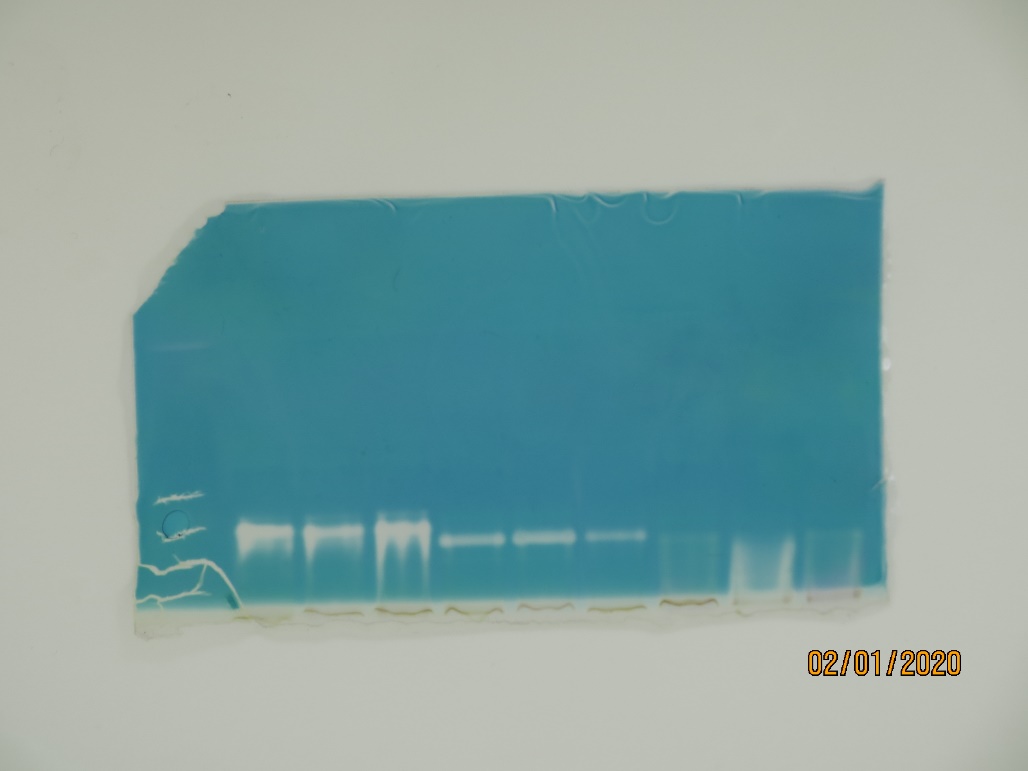


**B**

**
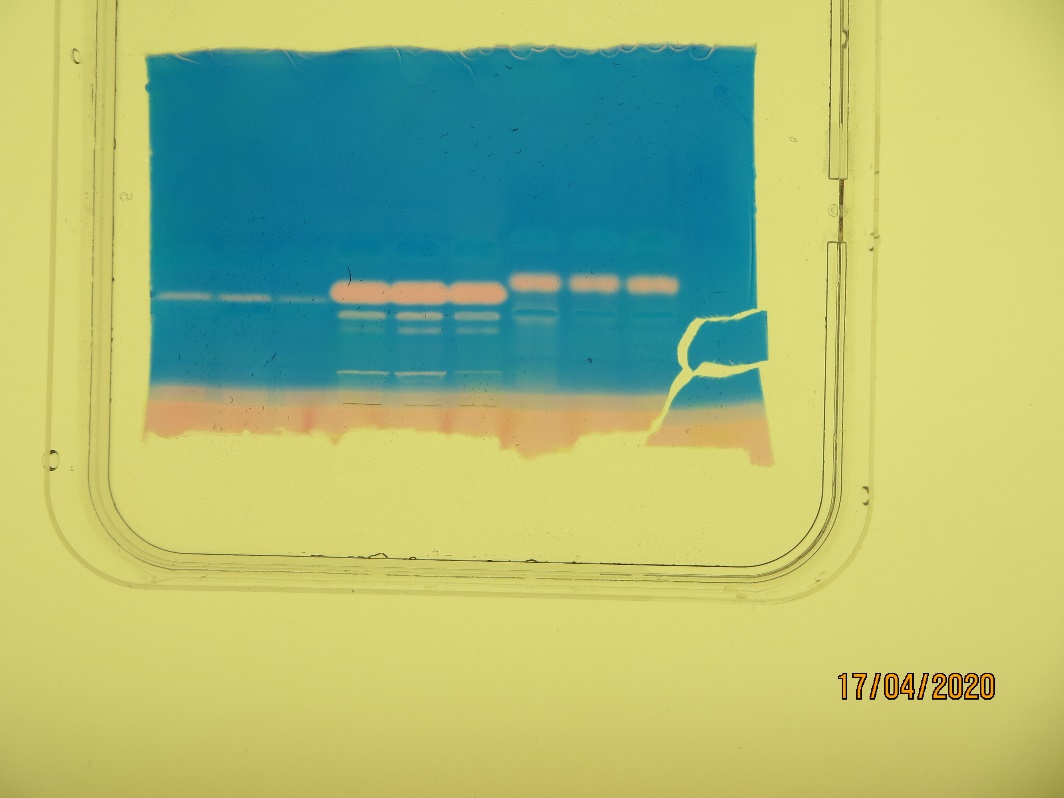
**

**S figure 2: HYAL1 activity measured using native zymography (full-length gels) in mouse, NMR and GP (from left to right) lymph nodes(A) and serum(B)**
